# Automated model-based lesion tracking in CT: colorectal liver metastases as a use case for development and performance analysis

**DOI:** 10.1186/s41747-026-00750-x

**Published:** 2026-07-14

**Authors:** Nalan Karunanayake, Hao Yang, Pengfei Geng, Qiyang Li, Ping Yin, Richard Kinh Do, Lawrence H. Schwartz, Lin Lu, Binsheng Zhao

**Affiliations:** 1 Department of Radiology, Memorial Sloan Kettering Cancer Center, New York, NY USA; 2 Department of Radiology, Harbin Medical University Cancer Hospital, Harbin, China; 3 Department of Radiology, Peking University People’s Hospital, Beijing, China

**Keywords:** Colorectal neoplasms, Image interpretation (computer-assisted), Liver neoplasms, Longitudinal studies, Tomography (x-ray computed)

## Abstract

**Objective:**

To develop and evaluate an automated CT liver lesion-tracking algorithm that matches lesions over time, detects new metastases, and reports per‑lesion confidence to support response assessment.

**Materials and methods:**

The study included 87 adults with unresectable colorectal liver metastases (CRLM) who had baseline and 8-week follow-up contrast-enhanced CT. Three radiologists generated a consensus reference. We developed a machine learning-driven, automated model-based lesion tracking (Auto-MBT) that provides per-lesion matching confidence. Performance was compared with: (1) deformable registration + overlap; (2) deformable registration + Auto-MBT; and (3) affine registration + Auto-MBT. Analyses were stratified by lesion size (< 1 cm, 1–3 cm, overall) and count (≤ 5, 6–10, > 10 per scan), and the triage utility of confidence scores was assessed by blinded adjudication. A publicly available melanoma dataset was used for external testing.

**Results:**

On the CRLM test set (35 pairs), affine + Auto-MBT matched 458/464 lesions (precision/recall 99%), detected 28/30 new lesions (90%/93%), and identified 42/44 disappeared lesions (93%/96%). Overall matching F1 was 0.989; affine + Auto-MBT outperformed deformable + overlap (0.797) and deformable + Auto-MBT (0.959). Confidence scores were higher for radiologist-accepted matches than rejected matches (0.72 *versus* 0.54) supporting triage utility. On the external set (23 pairs), overall matching F1 was 0.994, affine + Auto-MBT outperformed deformable + overlap (0.768) and deformable + Auto-MBT (0.971).

**Conclusion:**

Auto-MBT accurately tracks CRLM and flags new lesions across sizes and counts, with per‑lesion confidence to triage, not replace, clinician review, enabling more comprehensive and accurate therapy response assessment.

**Relevance statement:**

The automated tracking framework accurately matched liver lesions, including new and subcentimeter lesions, improving longitudinal assessment.

**Key Points:**

- Manual tracking of colorectal liver metastases (CRLM) on routine CT images is time-consuming and subject to inter-observer variability.
- The algorithm demonstrates robust lesion‑level tracking performance (F1 > 0.9) in high lesion‑count CRLM, including matched, new, and disappeared lesions.
- Automated total-tumor tracking complements Response Evaluation Criteria in Solid Tumors‒RECIST by quantifying all lesions, extending evaluation beyond limited target metastases.

**Graphical Abstract:**

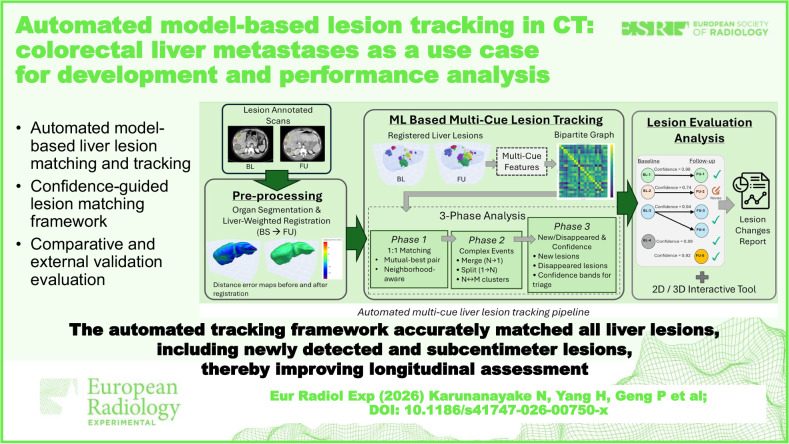

## Background

The liver is the most common site of solid tumor metastases, and longitudinal contrast-enhanced computed tomography (CT) is the standard for response assessment [1, 2]. Among cancers that metastasize to the liver, colorectal cancer is common. It is the third most frequent malignancy worldwide, and the liver is its predominant site of metastatic spread [3–5]. Colorectal liver metastases (CRLM) develop in up to 60% of patients with colorectal cancer, and at presentation, approximately 80% of those with stage IV disease have unresectable liver involvement [6–8]. Reliable lesion tracking over time is critical, yet assessment is largely manual, making comprehensive longitudinal review labor‑intensive and impractical when lesion counts are high [9, 10].

Under RECIST 1.1, radiologists measure up to two target lesions per organ, while remaining lesions are qualitatively categorized as non-target without formal measurement [11, 12]. Although non-target lesions are not specifically quantified, ignoring their size, volume, and morphologic change can obscure important patterns of treatment response, especially in multifocal disease [13]. This limitation is particularly relevant in liver metastases, where lesions vary widely in size, enhancement pattern, and shape due to therapy, motion, and heterogeneous vascular supply [14]. Existing automated lesion-tracking methods that are often based on deformable registration with spatial overlap heuristics struggle when lesion counts are high, lesions are small, morphologies change, or registration is imperfect [15–20]. More recent graph-based approaches improve spatial matching but still provide only binary match decisions without quantifying certainty, forcing radiologists to review all proposed correspondences with equal scrutiny. Few approaches account for biologically plausible complex changes such as lesion splits or mergers, further limiting their clinical utility.

To address these gaps, we developed an automated model-based lesion-tracking (Auto-MBT) system, a clinically oriented lesion-matching workflow that integrates established components of longitudinal imaging, including liver registration, multi-cue similarity modeling, and graph-based assignment, into a single system that outputs explicit lesion matches together with per-lesion confidence scores. In this study, we evaluate lesion-level tracking outcomes between two routine clinical time points, baseline and the first follow-up CT at approximately 8 weeks, in a challenging cohort of unresectable CRLM. Our primary objective is to quantify lesion-level matching performance (matched, new, and disappeared lesions), including in small-lesion and high-burden settings, and to assess whether confidence scores can support triage for clinician review, as a modular decision-support component that complements standard RECIST workflows by enabling scalable tracking across all measurable lesions. We further validated Auto-MBT on an external dataset of melanoma patients [21] and demonstrated its generalizability.

## Methods

### Ethics and regulatory compliance

This retrospective single-center study was approved by the Institutional Review Board of Memorial Sloan Kettering Cancer Center, with a waiver of informed consent granted due to the retrospective nature of the study. All procedures complied with the Health Insurance Portability and Accountability Act‒HIPAA and were conducted in accordance with the principles of the Declaration of Helsinki.

### Patient cohort selection

We retrospectively identified consecutive adults with unresectable CRLM treated with first-line chemotherapy at Memorial Sloan Kettering Cancer Center (2009–2019) who had baseline and ~8-week follow-up contrast-enhanced CT scans. First‑line treatment consisted of standard systemic regimens for CRLM (oxaliplatin‑ or irinotecan‑based combinations such as FOLFOX/XELOX or FOLFIRI/XELIRI, with or without anti‑VEGF/anti‑EGFR targeted agents), and a subset of patients received hepatic artery infusion pump chemotherapy. After applying exclusion criteria, which included incomplete liver coverage, non-contrast examinations, severe imaging artifacts, or substantial anatomical alterations (*e.g*., hepatic resection or advanced cirrhosis) that could preclude reliable longitudinal lesion tracking (full criteria are listed in Supplementary Material S1), 87 patients were included, contributing 174 scans (87 pairs). Accordingly, this pilot evaluation focuses on scan pairs (baseline *versus* first follow‑up) rather than multi‑timepoint trajectories across an entire treatment course.

### Imaging protocol and annotation

Portal-venous-phase contrast-enhanced CT scans were acquired on multidetector CT scanners as part of routine clinical care at Memorial Sloan Kettering Cancer Center. To document imaging heterogeneity, CT acquisition and reconstruction characteristics for both the internal Memorial Sloan Kettering Cancer Center cohort and the external melanoma cohort are provided in Supplementary Material Table S1. Three abdominal radiologists (5–10 years of post-training experience), blinded to clinical outcomes and to each other’s annotations, independently performed volumetric segmentation of all visible hepatic metastases on baseline and follow-up scan images using a customized Weasis medical viewer (version 2.0.7) [22]. At each follow‑up, lesions were cross‑referenced to baseline and labeled as matched, new (no baseline match), or disappeared (no follow‑up match) based on visual and morphologic criteria. For target-lesion analyses, the two largest measurable metastases per patient were selected from consensus masks (see Supplementary Material S2) and verified by a radiologist; remaining measurable lesions were considered non-target. All lesion masks used in this study were generated by manual radiologist segmentation, and no artificial intelligence-assisted lesion segmentation was used. No implanted radiopaque fiducial markers were present in or adjacent to hepatic lesions on the included baseline or follow‑up CT scans.

### Dataset partitioning

Patients were randomly assigned in a 60:40 ratio into a training cohort (*n* = 52 pairs) and an independent test cohort (*n* = 35 pairs) for final model evaluation (Table 1). The training set was used exclusively for algorithm development, while the independent test set was reserved for evaluation of the final model performance.

**Table 1:** Demographic characteristics, imaging data, and lesion annotation summary for the training and independent test cohorts (colorectal liver metastases dataset)

Characteristic	Training set (*n* = 52)	Test set (*n* = 35)	All patients (*n* = 87)
Demographics			
Age, median (IQR), years	62 (51–72)	58 (54–67)	61 (52–69)
Sex, male/female, *n* (%)	29/23 (55.8%/44.2%)	22/13 (62.9%/37.1%)	51/36 (58.6%/41.4%)
Lesion annotations			
Baseline lesions, total (RA-1/RA-2/RA-3)	-	332/267/287	991/807/874
Consensus, *n* (%)	558 (66.9%)	276 (33.1%)	834
Follow-up lesions, total (RA-1/RA-2/RA-3)	-	284/274/278	850/776/798
Consensus, *n* (%)	488 (65.1%)	262 (34.9%)	750
Lesion size distribution (consensus annotations)			
Baseline, *n* (%)			
< 1 cm	45	12	57 (6.8%)
1–3 cm	324	192	516 (61.9%)
> 3 cm	189	72	261 (31.3%)
Lesion diameter (mean ± SD)	2.91 ± 2.38	2.51 ± 1.86	2.77 ± 2.23
Number of lesions per scan (mean ± SD)	10.73 ± 7.45	7.88 ± 7.37	9.58 ± 7.55
Follow-up, *n* (%)			
< 1 cm	70	20	90 (12.0%)
1–3 cm	305	180	485 (64.7%)
> 3 cm	113	62	175 (23.3%)
Lesion diameter (mean ± SD)	2.33 ± 1.89	2.51 ± 1.74	2.39 ± 1.84
Number of lesions per scan (mean ± SD)	9.38 ± 6.92	7.48 ± 6.65	8.62 ± 6.84
Inter-reader agreement			
Baseline			86.49% 95% CI: [85.44%, 87.53%] *κ* = 0.762
Follow-up			86.25% 95% CI: [85.15%, 87.35%] *κ* = 0.753

Baseline and follow-up lesion counts are based on consensus annotations from three radiologists. Lesion size distribution is reported for consensus baseline and follow-up lesions, stratified by size category. Inter-reader agreement is calculated across all patients. RA = reader annotation from three abdominal radiologists (RA-1, RA-2, RA-3). Consensus annotations were determined by majority agreement among the three readers. Lesion size distribution is based on consensus annotations. Percentages in the “Consensus” rows represent the proportion of all annotated lesions agreed upon by all three readers. Inter-reader agreement (3 radiologists) calculated as percent complete agreement (95% CI) and Fleiss kappa ($\kappa$) for lesion detection in common patients (*n* = 87)

*CI* Confidence interval

### External validation

To test generalizability beyond CRLM, we applied the locked pipeline to the public longitudinal CT dataset (melanoma), which provides two imaging time points per patient (baseline and a post‑therapy follow‑up) with whole‑body portal‑venous CT and expert tumor masks [21]. Because the dataset includes multi‑organ tumor annotations, we restricted evaluation to hepatic lesions only. The liver was segmented using TotalSegmentator [23], and only lesion instances located within the liver were retained for analysis. All preprocessing, thresholds, and metrics were otherwise identical to the internal evaluation (see Supplementary Material S3). The 8‑week follow‑up interval criterion was specific to our internal CRLM cohort and was not imposed on the external melanoma dataset, in which follow‑up timing is determined by the source dataset acquisition schedule.

### Liver organ segmentation and affine alignment

As a preprocessing step, liver masks were generated with TotalSegmentator [23], refined, resampled to 1 × 1 × 1 mm, and used to crop both scans to the union bounding box. Follow-up was aligned to baseline *via* affine registration by maximizing normalized cross-correlation [24] on liver-mask distance maps, with craniocaudal weighting emphasizing the central liver to mitigate diaphragmatic variability (see Supplementary Material S4). This alignment preserved lesion geometry and was more robust than intensity-only methods, particularly under phase differences and high burden.

### Multi-cue lesion features

From lesion masks, we computed four cues per each pair of baseline and registered follow-up. First, spatial overlap measures the overlap coefficient between baseline and registered follow-up masks. Second, centroid proximity calculates the Euclidean proximity of lesion centroids. Third, mean-centered location assesses the distance between centroids after mean-centering within each scan, capturing relative position in liver space. Finally, nearest-neighbor shift reflects the similarity of local lesion density based on the change in each lesion’s nearest-neighbor distance (see Supplementary Material S5). These features were inputs to the multi-cue matching stage.

### Model development

Lesion correspondence across serial scans was established with a maximum-weight bipartite assignment. Baseline lesions and affine‑registered follow‑up lesions formed the two node sets of a bipartite graph, and candidate baseline–follow‑up pairs were edges weighted by the comprehensive multi‑cue similarity function integrating the extracted lesion features (see Supplementary Material S6.1). The maximum‑weight assignment selects a globally consistent set of one‑to‑one correspondences that maximizes total similarity across all selected pairs (rather than greedy matching), lesions without a selected counterpart are treated as provisional disappeared (baseline‑only) or new (follow‑up‑only) lesions prior to split/merge and general N ↔ M processing. Nearest-neighbor shift reflected local density to separate true disappearances from merges.

To handle small lesions, we used slight mask dilation for overlap checks, mutual-proximity safeguards, and size-adaptive priors based on clinical observations. Contextual validation further improved tracking by predicting expected lesion positions from neighboring lesion movements, particularly valuable for lesions with minimal direct overlap. In high-lesion-count scenarios with numerous metastases, the algorithm applied cluster detection (see Supplementary Material S6.2) to identify groups for coherent matching, enforced spatial compactness constraints for split/merge events, and detected hidden complex N-to-M relationships among spatially proximate lesions (see Supplementary Material S7).

Confidence estimation was performed separately for matched, disappeared, and new lesions using regularized logistic models with z-scored predictors (see Supplementary Material S6.3). For matched pairs, predictors captured multi-cue similarity strength, candidate margins, neighborhood consistency, spatial features, and registration quality. For unmatched lesions, predictors included maximum similarity to any candidate, local density change, entropy of alternative matches, and registration quality. Lesions were categorized into high (≥ 0.8), moderate (0.6–0.8), or low (< 0.6) confidence for radiologist triage. Because registration-quality features enter the confidence model, correspondences in scans with poorer alignment are expected to receive lower confidence and therefore be prioritized for review.

Cue weights and decision thresholds were tuned *via* Bayesian optimization (Optuna framework [25] with Tree-structured Parzen Estimator sampling [26]) to maximize lesion-level F1 score on the training set, with centroid-only match calculations used as a computational proxy during search to accelerate convergence. On an 8-core CPU system with 16 GB RAM and an NVIDIA A40 GPU, end-to-end processing per scan pair required approximately 3–5 min and was dominated by liver segmentation, while affine alignment and Auto-MBT matching completed in under 30 s.

### Performance metrics and statistical analysis

Performance was evaluated on the independent CRLM test set (*n* = 35), using radiologist consensus annotations as the reference, and on an external melanoma test set (*n* = 23). The primary outcome was lesion-level F1 score for matched lesions; secondary outcomes were precision and recall for matched, new, and disappeared lesion categories.

Pairwise algorithm comparisons were performed using patient-cluster bootstrap inference to account for multiple lesions per patient (patients/scan pairs as the resampling unit, 10,000 bootstrap replicates). For hypothesis testing, we prespecified lesion-level recall as the test statistic within each lesion category; therefore, *p*-values reported in Table 2 and Supplementary S6 correspond to recall comparisons. In each bootstrap replicate, lesion-level recall was recomputed for each algorithm, and pairwise differences in recall were used to estimate 95% confidence intervals and two-sided *p*-values (see Supplementary Material S8). By resampling at the patient level, inference reflects between-patient variability and avoids treating multiple lesions from the same patient as independent observations, preventing high lesion-burden patients from artificially narrowing confidence intervals or inflating significance. The *p*-values were adjusted for multiple comparisons using the Benjamini–Hochberg procedure, with *p* < 0.050 considered significant.

**Table 2:** Lesion-level precision, recall, and F1 score for the proposed affine registration + multi-cue tracking method *versus* two comparator approaches, with subgroup analysis by lesion size and total intrahepatic lesion count

Characteristic	Deformable + overlap	Deformable + auto-MBT	Affine + auto-MBT	BH‑adjusted *p*-value (recall; deformable + overlap *versus* deformable + auto-MBT)	BH‑adjusted *p*-value (recall; deformable + overlap *versus* affine + auto-MBT)
Overall performance					
Matched lesions				< 0.001	< 0.001
Precision	0.978 (0.955‒0.989)	0.984 (0.968‒0.992)	0.991 (0.978‒0.997)		
Recall	0.672 (0.628‒0.714)	0.935 (0.909‒0.954)	0.987 (0.972‒0.994)		
F1 score	0.797 (0.749‒0.837)	0.959 (0.936‒0.974)	0.989 (0.975‒0.995)		
Disappeared lesions				0.984	0.441
Precision	0.339 (0.259‒0.430)	0.727 (0.598‒0.827)	0.933 (0.821‒0.977)		
Recall	0.886 (0.760‒0.950)	0.909 (0.788‒0.964)	0.955 (0.849‒0.987)		
F1 score	0.491 (0.401‒0.581)	0.808 (0.685‒0.891)	0.944 (0.836‒0.974)		
New lesions				0.984	1.000
Precision	0.269 (0.193‒0.362)	0.643 (0.492‒0.770)	0.903 (0.751‒0.967)		
Recall	0.933 (0.787‒0.982)	0.900 (0.744‒0.965)	0.933 (0.787‒0.982)		
F1 score	0.418 (0.328‒0.514)	0.750 (0.602‒0.856)	0.918 (0.770‒0.974)		
Subgroup analysis					
By tumor count (F1 score)					
Matched					
≤ 5 lesions	0.905 (0.827‒0.950)	0.971 (0.918‒0.990)	1.000 (0.966–1.000)	0.042	< 0.001
6‒10 lesions	0.980 (0.892‒0.996)	1.000 (0.929–1.000)	1.000 (0.929–1.000)	NA	NA
> 10 lesions	0.716 (0.646‒0.777)	0.948 (0.916‒0.968)	0.984 (0.962‒0.993)	< 0.001	< 0.001
Disappeared					
≤ 5 lesions	0.167 (0.042‒0.476)	0.571 (0.213‒0.868) (*n* ≤ 10)	1.000 (*n* < 5)	NA	NA
6‒10 lesions	0.800 (*n* < 5)	1.000 (*n* < 5)	1.000 (*n* < 5)	NA	NA
> 10 lesions	0.507 (0.412‒0.602)	0.818 (0.687‒0.902)	0.938 (0.821‒0.981)	1.000	0.847
New					
≤ 5 lesions	0.182 (0.049‒0.492)	0.400 (*n* < 5)	1.000 (*n* < 5)	NA	NA
6‒10 lesions	0.889 (0.453‒0.987) (*n* ≤ 10)	1.000 (*n* < 5)	1.000 (*n* < 5)	NA	NA
> 10 lesions	0.404 (0.308‒0.507)	0.746 (0.580‒0.862)	0.902 (0.732‒0.969)	1.000	1.000
By lesion size (F1 score)					
Matched					
Small lesions (< 1 cm)	0.800 (0.571‒0.923)	0.981 (0.840‒0.998)	1.000 (0.875–1.000)	< 0.001	< 0.001
Small-to-medium lesions (≤ 3 cm)	0.793 (0.736‒0.840)	0.957 (0.928‒0.974)	0.989 (0.972‒0.996)	< 0.001	< 0.001
Disappeared					
Small lesions (< 1 cm)	0.727 (0.370‒0.924) (*n* ≤ 10)	1.000 (*n* < 5)	1.000 (*n* < 5)	NA	NA
Small-to-medium lesions (≤ 3 cm)	0.582 (0.479‒0.679)	0.860 (0.737‒0.931)	0.966 (0.863‒0.992)	0.984	0.441
New					
Small lesions (< 1 cm)	0.250 (0.066‒0.611)	0.667 (*n* < 5)	1.000 (*n* < 5)	NA	NA
Small-to-medium lesions (≤ 3 cm)	0.474 (0.371‒0.579)	0.776 (0.622‒0.880)	0.931 (0.780‒0.981)	0.984	1.000

The *p*-values correspond to patient-cluster bootstrap comparisons of lesion-level recall (patients/scan pairs as the resampling unit) with Benjamini–Hochberg (BH) adjustment. NA indicates < 5 patients contributed lesions in the stratum (inference not reported); “*n* ≤ 10” flags small sample size for cautious confidence interval (CI) interpretation

**Fig. 1:**
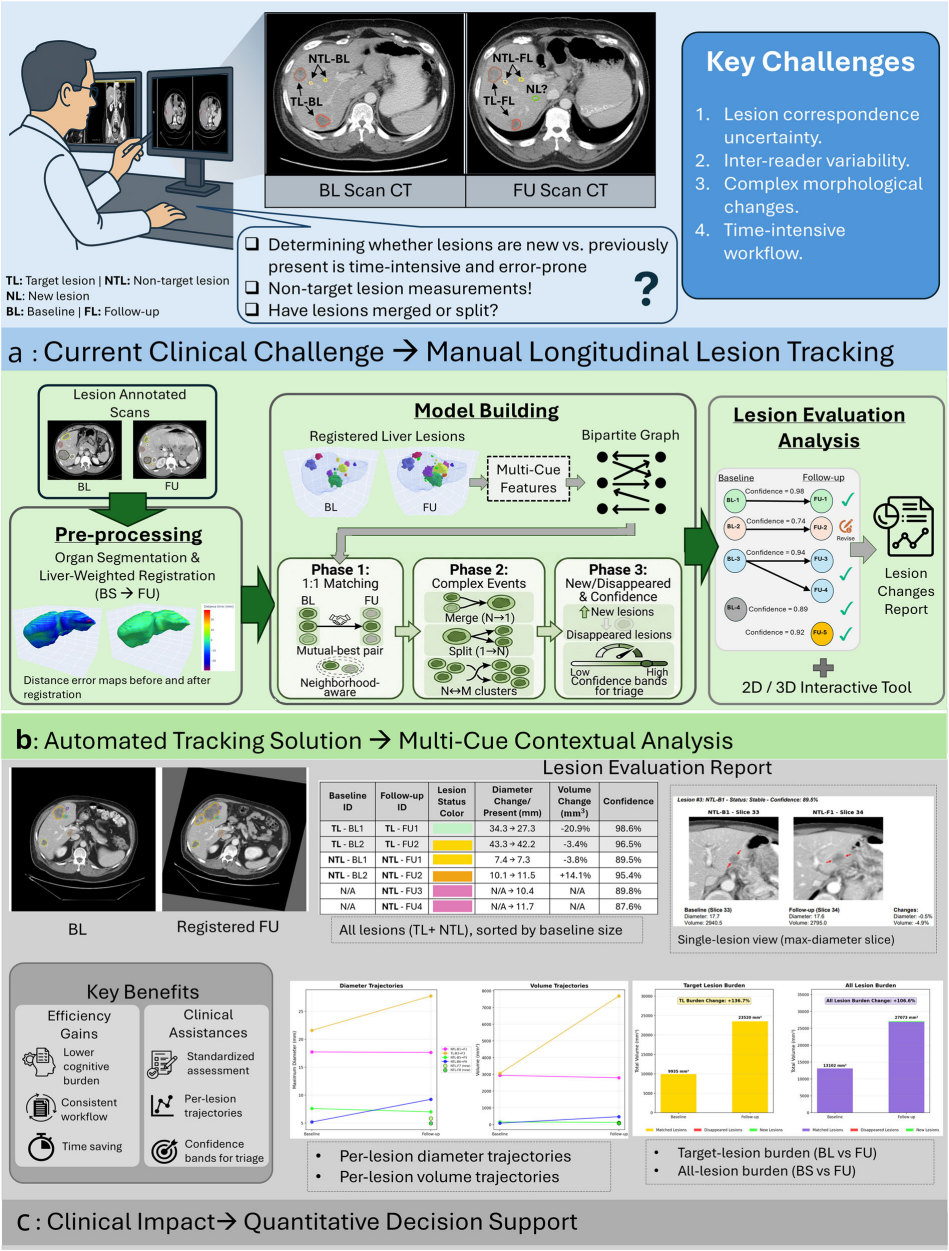
Overview of the proposed longitudinal lesion-tracking framework. **a** Manual tracking of colorectal liver metastases across serial CT scans is time-intensive, limited to a small number of target lesions, and subject to inter-reader variability, particularly in high-burden disease. **b** The proposed automated system applies affine liver-weighted registration, multi-cue feature extraction (including spatial, morphologic, and volumetric metrics with intra- and inter-timepoint neighborhood context), and graph-based bipartite matching to generate confidence-scored lesion correspondences. Complex events such as lesion split and merge are explicitly identified. **c** Outputs include two-dimensional (2D) and interactive three-dimensional (3D) lesion visualizations, confidence-based lesion evaluation reports, lesion-level trajectories, and whole-liver tumor burden metrics, supporting efficient, standardized, and explainable longitudinal assessment

The proposed system is a two-step pipeline combining affine registration with Auto-MBT, and was benchmarked against (1) deformable registration + overlap method [15, 20], and (2) deformable registration [15, 20] + Auto-MBT. For both deformable registration comparators, we used an intensity-based liver-masked affine with B‑spline deformable registration (see Supplementary Material S4). To account for clinical heterogeneity, results were stratified a priori by lesion size (< 1 cm, 0–3 cm, all lesions) and intrahepatic tumor count (≤ 5, 6–10, > 10 lesions/scan), thresholds commonly used in oncology practice (Fig. 1).

## Results

### Overall lesion-tracking performance

**Fig. 2:**
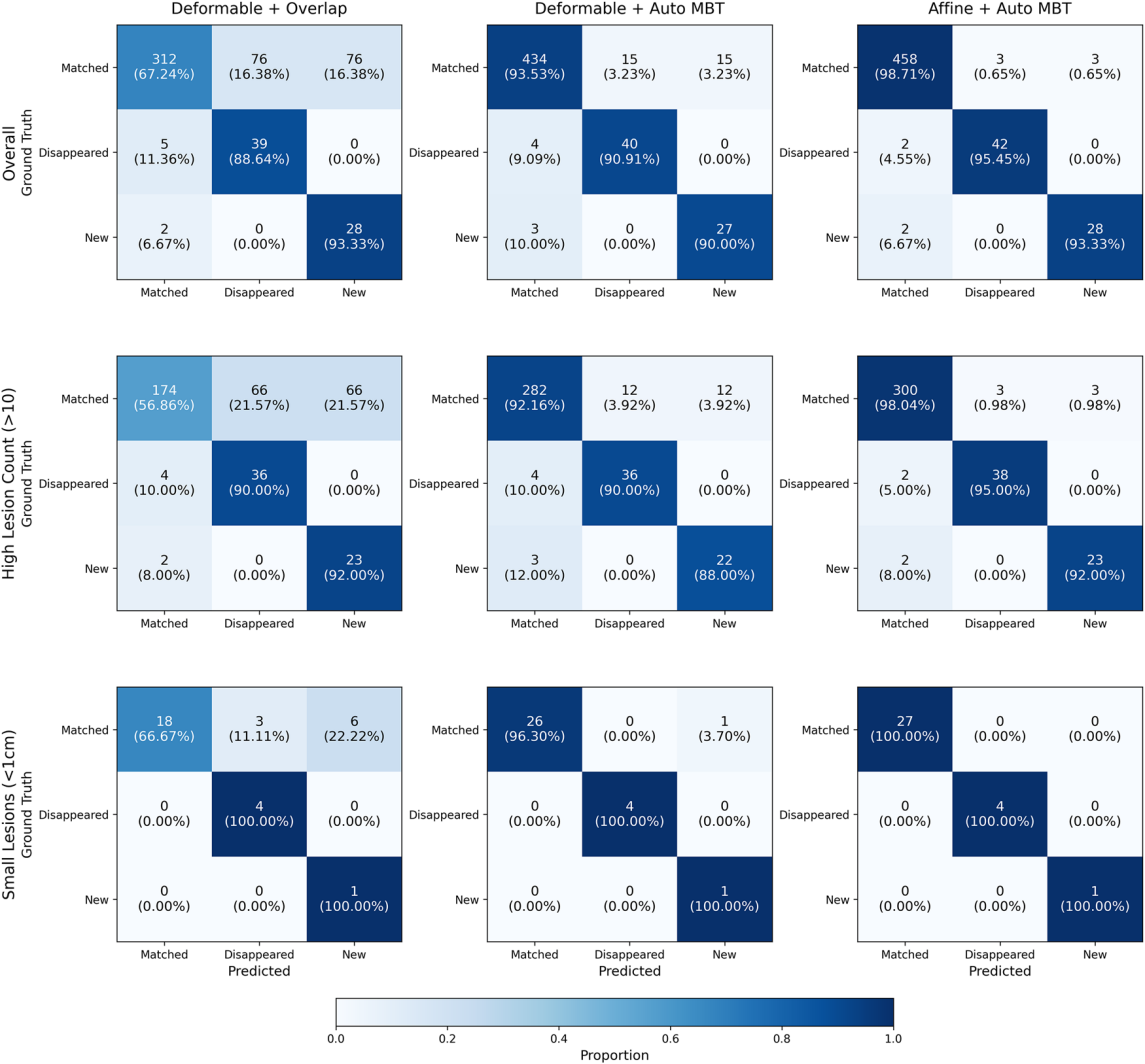
Confusion matrices summarize lesion-level tracking performance across three algorithms: (1) deformable + overlap, (2) deformable + Auto-MBT, and (3) affine + Auto-MBT (proposed). Rows denote ground truth (radiologist consensus); columns denote algorithm predictions. Performance is stratified across the full cohort, high tumor count cases (> 10 lesions), and small lesion cases (< 1 cm). Each cell reports the absolute count and percentage of lesions in the corresponding category. The proposed method demonstrates substantially fewer misclassifications across all conditions

The proposed affine + Auto-MBT achieved the highest accuracy for matched, new, and disappeared lesion classification in the independent CRLM test set (35 scan pairs, consensus reference) (Table 2, Fig. 2). For matched lesions, the F1 score was 0.989 (Table 2). Using matched‑lesion recall as the prespecified statistic for hypothesis testing, deformable + Auto‑MBT achieved significantly higher recall than deformable + overlap (*p* < 0.001). For new and disappeared lesions, affine + Auto-MBT also outperformed both alternatives (new: 0.918 *versus* 0.418/0.750, disappeared: 0.944 *versus* 0.491/0.808). On the external melanoma dataset (23 scan pairs), the same pattern held (Table S6, Fig. S2). Together, these results highlight two complementary gains: (1) multi-cue graph matching improves correspondence *versus* single-cue overlap matching; and (2) affine liver-weighted registration yields additional benefit over deformable registration, particularly for new and disappeared lesions.

Analysis of feature importance in the optimized model revealed distinct contributions to matching performance. While spatial overlap had the highest individual weight (0.548), the three complementary features of centroid proximity (0.300), mean-centered location (0.219), and nearest-neighbor shift (0.218) demonstrated that non-overlap features drive a substantial portion of matching decisions. This distributed feature importance, where multiple geometric and spatial cues together outweigh the primary overlap metric, explains why our multi-cue approach outperforms overlap-only baselines.

### Performance stratification by lesion size

The proposed model (affine + Auto-MBT) outperformed both comparators, deformable + Auto-MBT and deformable + overlap, across all lesion sizes (Table 2, Fig. 2). For lesions < 1 cm, it achieved matched F1 = 1.000 and F1 = 1.000 for disappeared and new lesions (*n* < 5 for these strata), compared with Deformable + Overlap (matched F1 = 0.800, new F1 = 0.250) and Deformable + Auto‑MBT (matched F1 = 0.981, new F1 = 0.667, *n* < 5). Hypothesis testing in Table 2 was performed on matched‑lesion recall using a patient/scan‑pair cluster bootstrap with Benjamini–Hochberg adjustment, in the < 1 cm stratum, recall differed significantly for the reported within‑family comparisons (*p* < 0.001). For lesions ≤ 3 cm, matched‑lesion recall again showed significant differences for the reported within‑family comparisons (*p* < 0.001) (Table 2). These gains were consistent across small lesions, including those < 1 cm. Similar result patterns were observed in the external melanoma dataset.

### Performance analysis by intrahepatic tumor count

Performance was evaluated across low (≤ 5 lesions per scan), intermediate (6–10), and high (> 10) intrahepatic lesion count (Table 2, Fig. 2).

In the low‑count group (≤ 5 lesions), Affine + Auto‑MBT achieved a strong matched‑lesion F1 = 1.000, compared with Deformable + Overlap (F1 = 0.905) and Deformable + Auto‑MBT (F1 = 0.971). Hypothesis testing was prespecified on matched‑lesion recall and performed using a patient‑level cluster bootstrap with Benjamini–Hochberg adjustment. In this subgroup, recall was significantly higher for Auto‑MBT than overlap within the deformable family (*p* = 0.042) and within the affine family (*p* < 0.001) (Table 2).

In the high‑count group (> 10 lesions), matched‑lesion performance remained robust for Affine + Auto‑MBT (F1 = 0.984), exceeding Deformable + Overlap (0.716) and Deformable + Auto‑MBT (0.948). Recall‑based bootstrap testing again demonstrated compelling improvement of Auto‑MBT over overlap within both the deformable and affine families (*p* < 0.001). In this group, Affine + Auto‑MBT also achieved higher F1 for new (0.902) and disappeared lesions (0.938) than both comparators (Table 2).

Confusion matrices (Fig. 2) further illustrate these trends, showing that in high-count cases, deformable + overlap model frequently misclassified matched lesions as disappeared (≈ 22%), and deformable + Auto-MBT reduced this error to ≈ 4%. Affine + Auto-MBT nearly eliminated such misclassifications (< 1%), while also improving detection of new and disappeared lesions. The external melanoma dataset displayed comparable result patterns (see Supplementary Material S10).

### Comparative analysis of algorithm performance *versus* individual radiologist performance

Against the consensus reference standard, affine + Auto-MBT outperformed all three individual radiologists across matched, new, and disappeared lesion categories (Table 3).

**Table 3:** Lesion-level precision, recall, and F1 score for the proposed affine + Auto-MBT and three individual radiologists, compared against consensus reference annotations

Category	Radiologist 1	Radiologist 2	Radiologist 3	Affine + auto-MBT
Matched lesions				
Precision	0.952 (0.928‒0.968)	0.937 (0.911‒0.955)	0.944 (0.919‒0.962)	0.991 (0.978‒0.997)
Recall	0.935 (0.909‒0.954)	0.955 (0.932‒0.970)	0.912 (0.882‒0.934)	0.987 (0.972‒0.994)
F1 score	0.943 (0.918‒0.961)	0.946 (0.921‒0.963)	0.928 (0.900‒0.948)	0.989 (0.975‒0.995)
Disappeared lesions				
Precision	0.989 (0.979‒0.995)	0.714 (0.549‒0.837)	0.633 (0.493‒0.753)	0.933 (0.821‒0.977)
Recall	0.773 (0.630‒0.872)	0.568 (0.422‒0.703)	0.705 (0.558‒0.818)	0.955 (0.849‒0.987)
F1 score	0.723 (0.581‒0.832)	0.633 (0.474‒0.767)	0.667 (0.522‒0.785)	0.944 (0.836‒0.974)
New lesions				
Precision	0.562 (0.393‒0.718)	0.633 (0.455‒0.781)	0.439 (0.299‒0.590)	0.903 (0.751‒0.967)
Recall	0.600 (0.423‒0.754)	0.543 (0.382‒0.695)	0.600 (0.423‒0.754)	0.933 (0.787‒0.982)
F1 score	0.581 (0.405‒0.738)	0.585 (0.414‒0.737)	0.507 (0.347‒0.666)	0.918 (0.770‒0.974)
Subgroup analysis				
High tumor count (> 10 lesions) (F1 score)				
Matched	0.938 (0.905‒0.960)	0.928 (0.894‒0.952)	0.920 (0.883‒0.945)	0.984 (0.962‒0.993)
Disappeared	0.741 (0.588‒0.851)	0.629 (0.461‒0.770)	0.709 (0.553‒0.827)	0.938 (0.821‒0.981)
New	0.638 (0.436‒0.801)	0.596 (0.396‒0.768)	0.519 (0.337‒0.695)	0.902 (0.732‒0.969)
Small lesions (< 1 cm) (F1 score)				
Matched	0.889 (0.626‒0.975)	0.875 (0.631‒0.966)	0.867 (0.621‒0.963)	1.000 (0.875–1.000)
Disappeared	NA	NA	NA	1.000 (*n* < 5)
New	0.769 (0.381‒0.948) (*n* ≤ 10)	0.571 (*n* < 5)	0.600 (0.231‒0.882) (*n* ≤ 10)	1.000 (*n* < 5)

Data are lesion-level performance metrics (95% confidence interval) for matched, disappeared, and new lesion classification. Consensus reference annotations were derived from the majority agreement among the three abdominal radiologists. Each radiologist’s performance was calculated against this consensus, excluding their own annotations from the reference set (leave-one-out)

*NA* Not applicable due to insufficient sample size for reliable estimation

For matched lesions, the algorithm achieved an F1 score of 0.989 (95% confidence interval 0.975–0.995) *versus* 0.928–0.946 for radiologists. Disappeared-lesion detection reached 0.944 *versus* 0.633–0.723, and new-lesion detection 0.918 *versus* 0.507–0.585. Performance advantages were most pronounced in high lesion-count (> 10 lesions/scan) and < 1 cm lesion subgroups, where the algorithm achieved F1 scores up to 1.000 compared with 0.867–0.938 for radiologists.

These results show that the algorithm not only matches but consistently exceeds radiologist accuracy, with the largest relative improvements occurring in high-burden and small-lesion scenarios.

### Complex lesion evolution

**Fig. 3:**
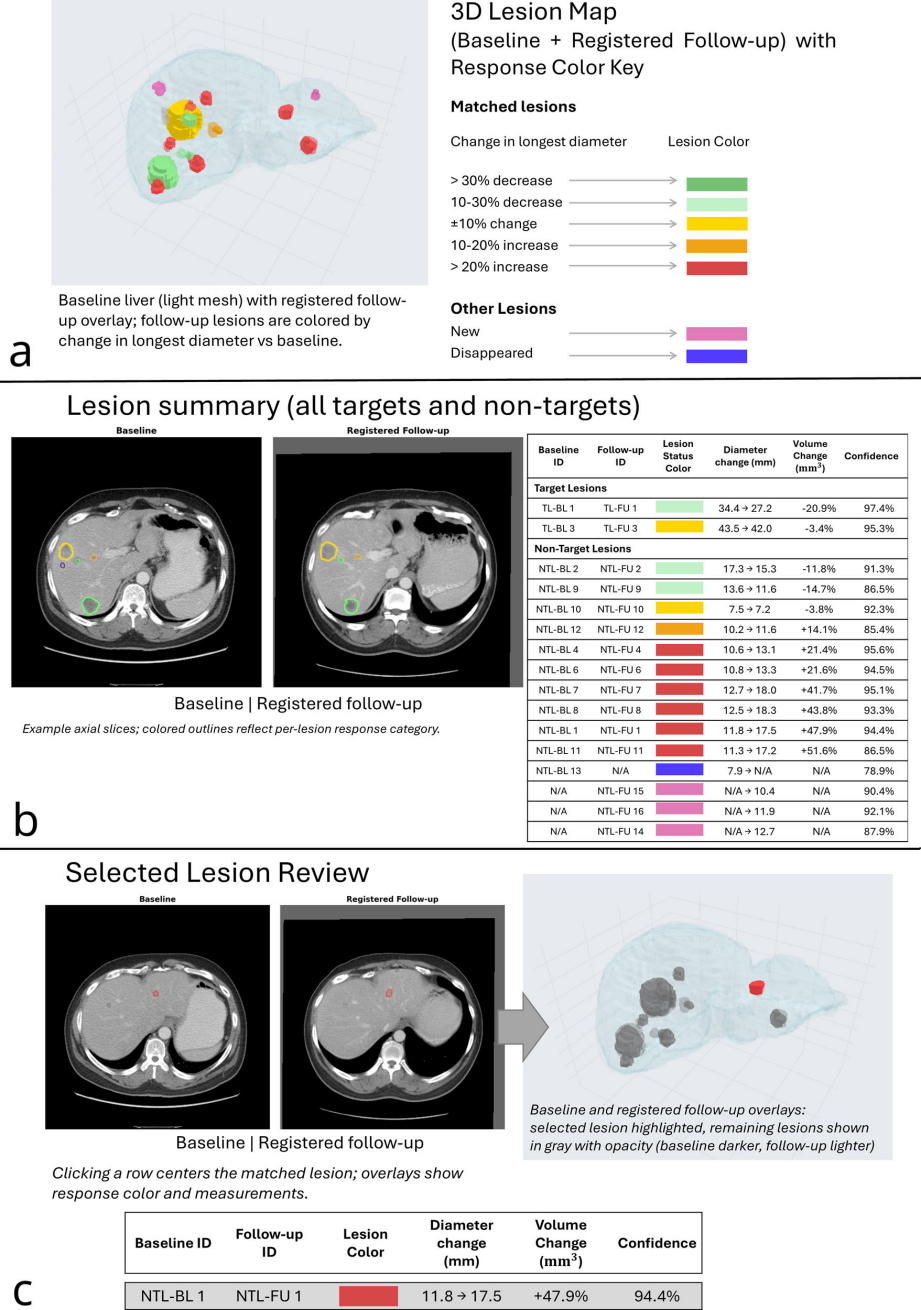
Interactive review for longitudinal lesion tracking. **a** Three-dimensional (3D) overview with baseline liver as a light mesh and registered follow-up overlaid; follow-up lesions are color-coded by per-lesion change (ranges shown in the panel legend). **b** Synchronized two-dimensional (2D) review displays baseline and registered follow-up axial images; the accompanying table lists all lesions (targets and non-targets) with identifiers, response color, diameter/volume change, and a confidence score (thresholds indicated in the panel). **c** Selected-lesion view: choosing a lesion (matched pair) centers the two slices and highlights the lesion in color; a compact metrics strip reports the longest diameter and volume at both time points with % change, and a 3D locator shows its position while other lesions are grayed

**Fig. 4:**
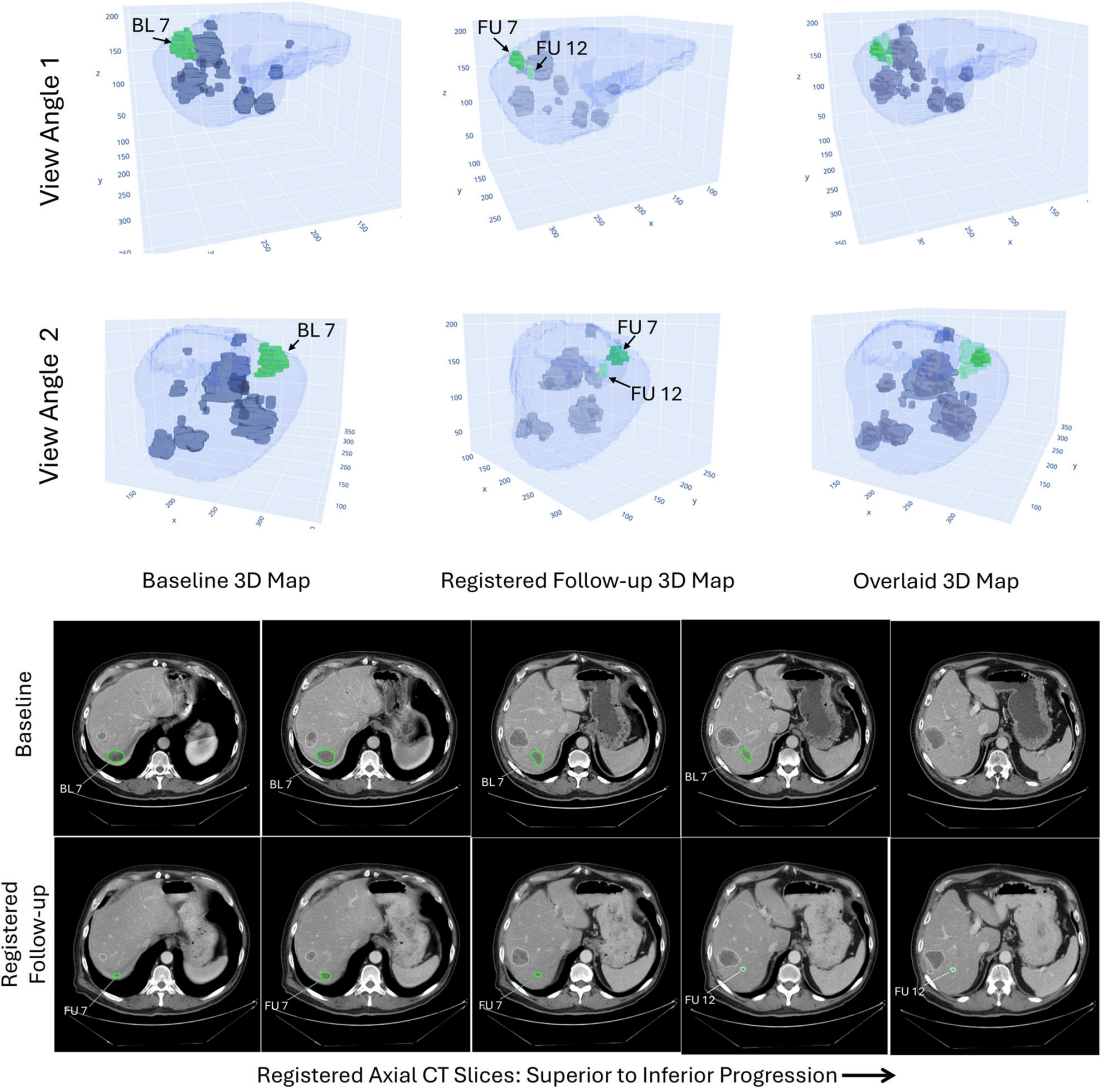
Complex lesion evolution: split illustrated in three-dimensional (3D) and two-dimensional (2D) views. (**Top**) Two viewpoints show the baseline 3D map (single parent lesion BL7), the registered follow-up 3D map (daughter lesions FU7 and FU12), and the overlaid map demonstrating spatial continuity and local neighborhood preservation after affine liver-weighted registration. (**Bottom**) Registered axial slices (superior - inferior) depict geometric separation of the daughter lesions, consistent with the 3D overlay; colors follow the response scheme in Fig. 3. The algorithm classified this correspondence as a split (1 → 2) using multi-cue matching with neighborhood/compactness constraints; the approach generalizes to N ↔ M relationships (see Supplementary Fig. S4)

The interactive interface (Fig. 3) presents all lesions with a standardized response color scheme and per-lesion confidence, linked to synchronized baseline and registered follow-up axial images and a three-dimensional locator. Selecting a lesion either *via* the table or directly on the slice recenters the two- and three-dimensional views and displays lesion-level longest-diameter and volume changes with their confidence scores. Quantitative summaries of lesion trajectories and target-lesion *versus* all-lesion burden are provided in Supplementary Fig. S3. Beyond 1-1 matches, the algorithm resolves non-bijective correspondences using neighborhood context and spatial compactness, identifying split (1 → N), merge (N → 1), and general N ↔ M relationships (Fig. 4, Supplementary Fig. S4).

### Confidence-based lesion-tracking accuracy and triage utility

**Fig. 5:**
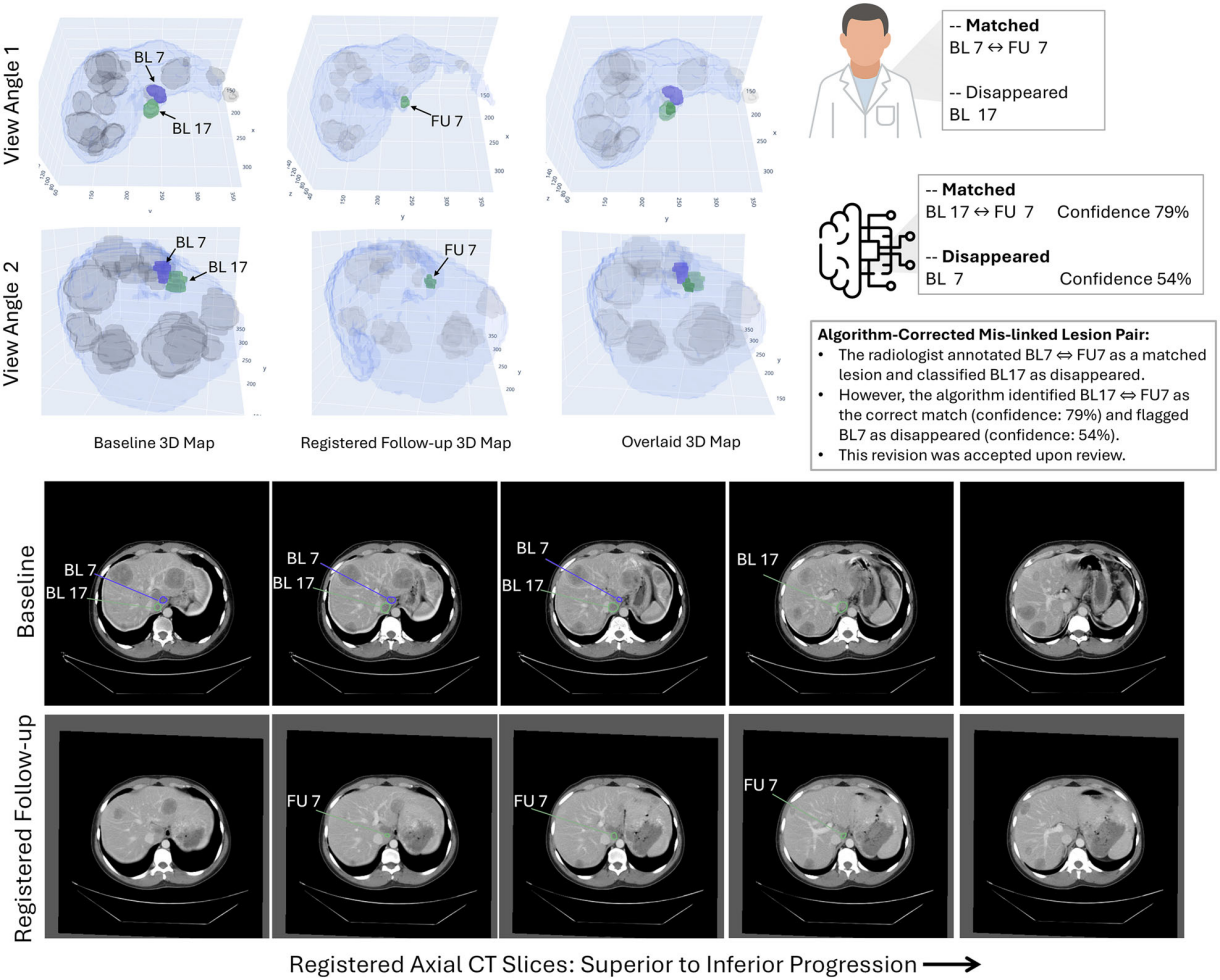
Confidence-informed review of radiologist annotations in high–lesion-count colorectal liver metastases. (**Top**) Baseline, registered follow-up, and overlaid three-dimensional (3D) liver maps from two viewpoints. (**Bottom**) Registered axial slices confirm the corrected correspondence highlighted by the algorithm. Confidence scores support targeted review in dense, multifocal disease

Across the independent CRLM test set, the algorithm generated 58 discrepancy alerts, with mis-linked alerts occurring in 3.8%, 1.2%, and 4.3% of lesions for Readers 1, 2, and 3, respectively. This indicates that alerts were infrequent relative to the total number of lesions reviewed. Most alerts were accepted after blinded adjudication (49/58, 84.5%, 95% confidence interval 73.0–91.6). Accepted alerts had higher confidence than rejected alerts by a median difference of 0.181 (0.720 [0.577–0.821] *versus* 0.541 [0.395–0.595], *p* = 0.023), indicating that the score is predictive and suitable for triage (Table 4, Fig. 5). By alert type, acceptance was 79.2% for mis-linked pairs (median difference 0.215, *p* = 0.049), 82.4% for false disappearances (median difference 0.393, *p* = 0.006), and 94.1% for false new lesions (median difference -0.090, *p* = 0.588, few rejections). Per-reader patterns were similar (*e.g*., for Reader 3, mis-linked pairs showed a median difference of 0.361, *p* = 0.037), with *p*-values not computed in strata where one group was empty (Table 4).

**Table 4:** Algorithm-flagged discrepancy alerts and blinded radiologist adjudication outcomes, with confidence summaries (per reader and aggregate)

Reader/class	Alerts/lesions reviewed	Lesions reclassified	Accepted after revision (%) [95% CI]	Confidence (accepted), median [IQR]	Confidence (rejected), median [IQR]	Δ-confidence	*p*-values
Radiologist 1							
Mis-linked lesion pair	10/263	N = 4 D = 4 MO = 2	90.0 [59.6–98.2]	0.718 [0.550–0.781]	0.519 [0.519–0.519]	+0.199	0.159
False disappearance	7/59	NA	85.7 [48.7–97.4]	0.690 [0.624–0.715]	0.285 [0.285–0.285]	+0.405	0.286
False new lesion	7/21	NA	85.7 [48.7–97.4]	0.807 [0.563–0.922]	0.866 [0.866–0.866]	-0.070	0.857
Radiologist 2							
Mis-linked lesion pair	3/254	N = 0 D = 1 MO = 2	100.0 [43.8–100.0]	0.715 [0.633–0.718]	-	-	-
False disappearance	5/13	NA	100.0 [56.6–100.0]	0.679 [0.677–0.746]	-	-	-
False new lesion	4/20	NA	100.0 [51.0–100.0]	0.758 [0.703–0.776]	-	-	-
Radiologist 3							
Mis-linked lesion pair	11/256	N = 3 D = 3 MO = 5	63.6 [35.4–84.8]	0.933 [0.878–0.952]	0.573 [0.548–0.645]	+0.361	0.037
False disappearance	5/31	NA	60.0 [23.1–88.2]	0.586 [0.583–0.635]	0.334 [0.303–0.365]	+0.252	0.200
False new lesion	6/22	NA	100.0 [61.0–100.0]	0.796 [0.206–0.861]	-	-	-
All							
All alert types	58/939	-	84.5 [73.0–91.6]	0.720 [0.577–0.821]	0.541 [0.395–0.595]	+0.181	0.023

Accepted = annotation revised after blinded review; 95% confidence intervals by Wilson; confidence as median [interquartile range]; Δ-confidence = median (accepted) - median (rejected); *p*-values by Mann–Whitney U (shown as “—” if one group is empty). Reclassification: N = new, D = disappeared, MO = matched to other; counts reported for mis-linked alerts; not tallied for false disappearance/new alerts. All = pooled across readers

**Fig. 6:**
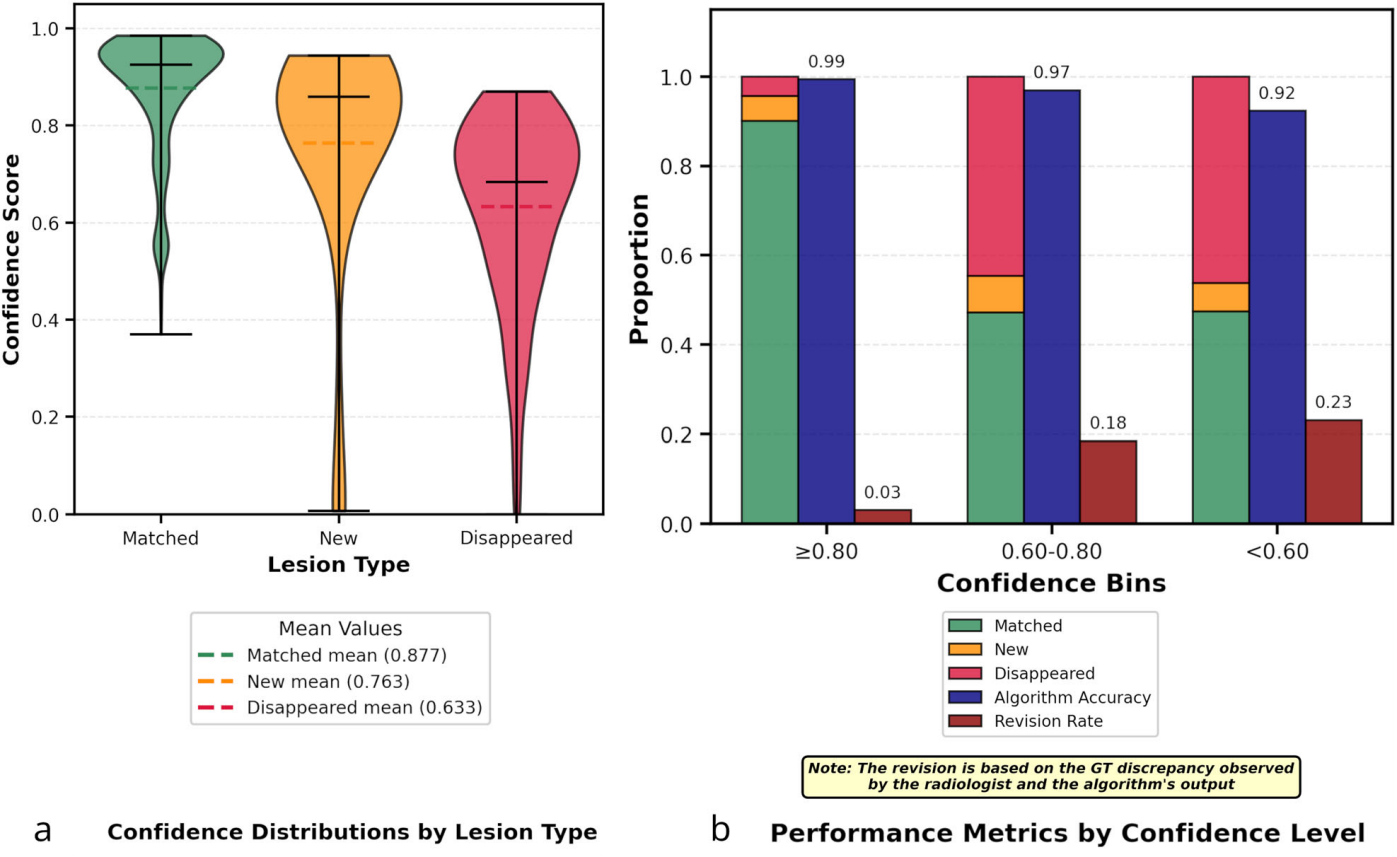
Confidence distributions and triage utility in high–lesion-count colorectal liver metastases. **a** Confidence score distributions by lesion class (matched, new, disappeared) in the high–lesion-count subset (> 10 lesions/scan). **b** Performance stratified by confidence bins

When confidence scores were analyzed in high tumor-count cases (> 10 lesions/scan), confidence distributions differed by lesion class (Fig. 6a), with matched lesions showing higher central confidence (mean = 0.877) than new (= 0.763) and disappeared (= 0.633) lesions, consistent with their greater detection difficulty.

Stratifying performance metrics by confidence thresholds (Fig. 6b) yielded a monotonic pattern where for thresholds ≥ 0.80, accuracy was 0.99 with revision 0.03, while for thresholds 0.60–0.80, accuracy was 0.97 with revision 0.18, and for thresholds < 0.60, accuracy was 0.92 with revision 0.23. Low-confidence lesions comprised fewer cases but showed greater uncertainty. This confidence-graded separation of accuracy and revision supports a triage strategy in which high-confidence correspondences undergo minimal oversight, while lower-confidence cases are prioritized for closer inspection.

## Discussion

This study presents an automated CT lesion-tracking system (Auto-MBT) that evaluates all measurable lesions and reports per-lesion confidence estimates. We evaluated the approach on a consecutive cohort of adults with unresectable CRLM imaged at baseline and 8-week follow-up, as well as an external longitudinal dataset with liver lesions from melanoma patients. The model achieved robust lesion-level accuracy for matched, new, and disappeared lesions and outperformed both a deformable + overlap baseline and deformable + Auto-MBT hybrid models. The largest margins appeared precisely where clinical reading is hardest, specifically in small lesions and high–lesion-count studies.

Two design choices underlie these gains. First, liver-masked weighted affine registration on distance maps preserves lesion geometry while stabilizing the organ frame, reducing local warping errors that disproportionately affect small and adjacent lesions. Second, multi-cue matching improves robustness over overlap-only rules with a coherent correspondence model that integrates spatial overlap, centroid proximity, mean centroid location, and neighborhood context across time points. The comparator analysis isolates both effects. Substituting multi-cue matching for overlap-only improves correspondence, and substituting liver-masked affine registration for deformable alignment provides further benefits, particularly for new and disappeared lesions where spatial stability is critical. Unlike existing overlap-only methods that fail when spatial correspondence is ambiguous, our multi-cue framework leverages centroid proximity (23%), anatomical location (17%), and neighborhood context (17%) alongside spatial overlap (42%), reflecting their relative contributions. This multi-feature approach enabled robust matching even when overlap was minimal, explaining our method’s 99% accuracy for small lesions compared to 80% for overlap-only approaches, and robust performance in high-burden cases where spatial features alone proved insufficient.

Clinically, this workflow supports a shift from target-only sampling to all-lesion tracking, enabling radiologists to review individual lesion change even when lesion counts are high. In routine follow-up of CRLM, most measurable lesions are not formally measured. Here, every measurable lesion is matched and tracked across the baseline and the first follow-up, and lesion-level changes (diameter, volume, emergence, and disappearance) are summarized alongside target-lesion metrics. This whole-liver view does not replace RECIST. Target-lesion sum of longest diameters and new-lesion rules remain applicable. Rather, it complements RECIST by documenting disease heterogeneity and non-target changes that may presage failure or response. In head-to-head comparison with individual radiologists against a consensus reference, the algorithm showed higher lesion-level performance, with the clearest margins in high-count (> 10 lesions/scan) cases, supporting its role as a confidence-informed second reader where manual tracking is most error-prone (Table 3).

A central contribution is the move beyond binary match/no-match to a confidence-scored, transparent output. Confidence behaved predictably by task difficulty and stratified real-world behavior, with accuracy rising and revision burden falling monotonically with higher confidence in prespecified bands of < 0.60, 0.60–0.80, and ≥ 0.80. In blinded discrepancy adjudication, alerts ultimately accepted by readers carried higher confidence than those they rejected, indicating that the score reflects case difficulty rather than echoing user bias. Practically, this supports a triage policy where ≥ 0.80 correspondences can pass with minimal oversight, 0.60–0.80 warrant focused review, and < 0.60 are prioritized for full adjudication.

Longitudinal evolution in CRLM is frequently non-bijective. By enforcing spatial compactness and neighborhood consistency, the method identifies split (1 → N) and merge (N → 1) events and general N ↔ M mappings, then surfaces them for review in synchronized three-dimensional overlays and axial slices. Making these events explicit helps prevent false progression/regression calls that arise when multiple nearby lesions are paired incorrectly or when a confluent follow-up lesion is mistaken for a disappearance.

For adoption, we built a visual interface that pairs analytics with readable outputs. The tool generates a concise lesion-level report (identifiers, response color, percentage changes, and confidence), includes zoomed snapshots of the maximal-diameter slice, and provides a three-dimensional locator for spatial context (see Supplementary Material S11). Outputs can be exported as secondary-capture objects suitable for Picture Archiving and Communication System‒PACS and tumor-board workflows, allowing review without custom viewers. This standardization also supports trainee supervision and Quality Assurance audits in high lesion-count studies.

We describe representative failure cases in Supplementary Material S12. Errors most often occur with clustered lesions, lesions near the liver boundary, and hypervascular lesions with variable enhancement or interval morphologic change, which radiologists can often resolve using local anatomic landmarks. Affine alignment can also be suboptimal under extreme deformation, including postoperative change, large confluent lesions, or marked interval morphologic change. Although registration quality is incorporated into the confidence score to flag uncertain cases for review, additional constrained refinement strategies may further improve robustness.

Auto-MBT is not intended to redefine RECIST rules. It aims to make the longitudinal review underlying RECIST more complete and internally consistent. While improved correspondence could influence borderline situations (*e.g*., confirming a truly new lesion or correcting an apparent disappearance due to a mismatch), final response categorization remains governed by standard RECIST criteria.

While the Auto-MBT results are promising, several limitations should be acknowledged. First, this is a retrospective single-institute study focused on portal-venous-phase CT with an approximately 8-week interval, and evaluations were based on scan pairs (baseline *versus* first follow-up). Sequential pairwise application across extended follow-up intervals may introduce cumulative error. Second, the test cohort size is limited. Subgroup sample sizes were small for certain analyses (disappeared/new in specific size/count strata). Third, this study used manual lesion masks to establish a robust reference for algorithm development and evaluation. Full clinical deployment will require integration with automated segmentation [27, 28] and prospective validation, although Auto-MBT is designed to tolerate imperfect masks *via* multi-cue matching and confidence scoring. Finally, evaluation remains confined to hepatic lesions on contrast-enhanced CT. We did not explicitly evaluate mixed contrast phases or systematically stratify performance by reconstruction kernel or dose settings.

Future work will focus on multi-institutional evaluation of Auto-MBT using large, multi-timepoint datasets acquired under diverse imaging protocols (*e.g*., slice thickness and spacing, in-plane resolution, reconstruction kernel/algorithm, and contrast enhancement) and across multiple anatomic sites, to evaluate real-world robustness to acquisition variability and to quantify downstream decision impact. Furthermore, future work will focus on translating the proposed lesion‑level correspondence module into an end‑to‑end longitudinal response assessment pipeline that integrates automated lesion detection/segmentation [27, 28], tracking with confidence‑guided triage, target lesion selection, longitudinal measurement propagation, and automated RECIST 1.1 response categorization. Because Auto‑MBT is modular, it can be readily combined with existing measurement and reporting systems without retraining the full pipeline, and augmented with routine confidence calibration checks to ensure reliability across sites and scanners.

In addition, prospective multi‑reader studies will quantify whether confidence‑guided correspondence review meaningfully changes RECIST 1.1 response assignments, improves consistency and efficiency, and reduces unnecessary adjudication in clinical workflows. Finally, workflow studies will assess human–artificial intelligence interaction (*e.g*., how confidence thresholds affect reading time, correction rates, and user trust) and compare performance, where feasible, against deployed commercial or regulatory-cleared longitudinal assessment tools.

## Supplementary information

**Additional file 1: Table S1.** CT acquisition and reconstruction characteristics for the internal MSKCC cohort and the external melanoma cohort. **Table S2.** Lesion annotation summary for the external validation dataset (melanoma patients, liver lesions). **Table S3.** Predictors for matched pair confidence. **Table S4.** Predictors for lonely lesion confidence. **Table S5.** Thresholds for Complex Event Identification. Table S6. Lesion-Level Precision, Recall, and F1-Score for the Proposed Affine + Auto MBT Versus Two Comparator Approaches, with Subgroup Analysis by Lesion Size and Total Intrahepatic Lesion Count (external melanoma dataset). **Fig. S1.** Auto-MBT model development pipeline. **Fig. S2.** Confusion matrices summarize lesion-level tracking performance across three algorithms (external melanoma dataset): (1) deformable + overlap, (2) deformable + Auto MBT, and (3) affine + Auto MBT (proposed). Rows denote ground truth (radiologist consensus); columns denote algorithm predictions. Performance is stratified across the full cohort, high tumor count cases (>10 lesions), and small lesion cases (<1 cm). Each cell reports absolute count and percentage of lesions in the corresponding category. The proposed method demonstrates substantially fewer misclassifications across all conditions. **Fig. S3.** (Top) Quantitative summaries plot lesion-level trajectories (longest diameter and volume) and compare baseline versus follow-up burden for target lesions and (bottom) for all lesions combined. Color categories are for visualization. **Fig. S4.** Complex lesion evolution—Merge illustrated in 3D and 2D. (Top) Two viewpoints show the baseline 3D map (multiple parent lesions BL10, BL19, BL20, BL22), the registered follow-up 3D map (matched lesion FU10), and the overlaid map demonstrating spatial continuity and preserved lesion neighborhood after affine liver-weighted registration. (Bottom) Registered axial CT slices (superior–inferior) depict the geometric correspondence between baseline and follow-up lesions, consistent with the 3D overlay; colors follow the response scheme in Fig 3. **Fig. S5.** Auto-MBT failure case 1: ID‑switch in clustered pericaval lesions. **Fig.S6.** Auto-MBT failure case 2: ID‑switch for boundary‑adjacent lesions under residual misregistration. **Fig. S7.** Auto-MBT failure case 3: Missed correspondence with false “disappeared/new” labels under marked interval deformation.

## Data Availability

The internal dataset used in this study is not publicly available due to institutional restrictions. However, de-identified data and tumor annotations may be shared upon reasonable request to the corresponding author, subject to institutional approval.

## Abbreviations

Auto-MBT: Automated model-based lesion tracking
CRLM: Colorectal liver metastases
CT: Computed tomography
RECIST: Response Evaluation Criteria in Solid Tumors

## References

[1] Chen W, Hoffmann AD, Liu H, Liu X (2018) Organotropism: new insights into molecular mechanisms of breast cancer metastasis. NPJ Precis Oncol 2:4. 10.1038/s41698-018-0047-0 29872722 10.1038/s41698-018-0047-0 PMC5871901

[2] Obenauf AC, Massagué J (2015) Surviving at a distance: organ-specific metastasis. Trends Cancer 1:76–91. 10.1016/j.trecan.2015.07.009 28741564 10.1016/j.trecan.2015.07.009 PMC4673677

[3] Sung H, Ferlay J, Siegel RL et al (2021) Global cancer statistics 2020: GLOBOCAN estimates of incidence and mortality worldwide for 36 cancers in 185 countries. CA Cancer J Clin 71:209–249. 10.3322/caac.21660 33538338 10.3322/caac.21660

[4] Siegel RL, Giaquinto AN, Jemal A (2024) Cancer statistics, 2024. CA Cancer J Clin 74:12–49. 10.3322/caac.21820 10.3322/caac.21820 38230766

[5] Li J, Pan J, Wang L et al (2025) Colorectal cancer: pathogenesis and targeted therapy. MedComm 6:e70127. 10.1002/mco2.70127 10.1002/mco2.70127 PMC11885891 40060193

[6] Nardo B, Serafini S, Ruggiero M et al (2016) Liver resection for metastases from colorectal cancer in very elderly patients: new surgical horizons. Int J Surg 33:S135–S141. 10.1016/j.ijsu.2016.06.014 27353843 10.1016/j.ijsu.2016.06.014

[7] Mohammad WM, Martel G, Mimeault R et al (2012) Evaluating agreement regarding the resectability of colorectal liver metastases: a national case-based survey of hepatic surgeons. HPB (Oxford) 14:291–297. 10.1111/j.1477-2574.2012.00440.x 22487066 10.1111/j.1477-2574.2012.00440.x PMC3384848

[8] Leone N, Arolfo S, Spadi R et al (2023) Colorectal cancer with synchronous unresectable liver metastases: resecting the primary tumor improves survival. Int J Colorectal Dis 38:169. 10.1007/s00384-023-04469-8 37322315 10.1007/s00384-023-04469-8

[9] Wesdorp NJ, Zeeuw JM, Postma SCJ et al (2023) Deep learning models for automatic tumor segmentation and total tumor volume assessment in patients with colorectal liver metastases. Eur Radiol Exp 7:75. 10.1186/s41747-023-00383-4 38038829 10.1186/s41747-023-00383-4 PMC10692044

[10] Folio LR, Sandouk A, Huang J et al (2013) Consistency and efficiency of CT analysis of metastatic disease: semiautomated lesion management application within a PACS. AJR Am J Roentgenol 201:618–625. 10.2214/AJR.12.10136 23971455 10.2214/AJR.12.10136 PMC6771287

[11] Eisenhauer EA, Therasse P, Bogaerts J et al (2009) New response evaluation criteria in solid tumours: revised RECIST guideline (version 1.1). Eur J Cancer 45:228–247. 10.1016/j.ejca.2008.10.026 19097774 10.1016/j.ejca.2008.10.026

[12] Bucho TMT, Tissier RLM, Bodalal Z et al (2024) How does target lesion selection affect RECIST? A computer simulation study. Invest Radiol 59:465–471. 10.1097/RLI.0000000000001045 37921780 10.1097/RLI.0000000000001045

[13] Fournier L, de Geus-Oei L-F, Regge D et al (2022) Twenty years on: RECIST as a biomarker of response in solid tumours an EORTC imaging group—ESOI joint paper. Front Oncol 11:800547. 10.3389/fonc.2021.800547 10.3389/fonc.2021.800547 PMC8784734 35083155

[14] Chun YS, Vauthey J, Boonsirikamchai P et al (2009) Association of computed tomography morphologic criteria with pathologic response and survival in patients treated with bevacizumab for colorectal liver metastases. JAMA 302:2338–2344. 10.1001/jama.2009.1755 19952320 10.1001/jama.2009.1755 PMC4139149

[15] Szeskin A, Rochman S, Weiss S et al (2023) Liver lesion changes analysis in longitudinal CECT scans by simultaneous deep learning voxel classification with SimU-Net. Med Image Anal 83:102675. 10.1016/j.media.2022.102675 36334393 10.1016/j.media.2022.102675

[16] Di Veroli B, Lederman R, Shoshan Y et al (2024) A graph-theoretic approach for the analysis of lesion changes and lesions detection review in longitudinal oncological imaging. Med Image Anal 97:103268. 10.1016/j.media.2024.103268 39029156 10.1016/j.media.2024.103268

[17] Rochman S, Szeskin A, Lederman R et al (2024) Graph-based automatic detection and classification of lesion changes in pairs of CT studies for oncology follow-up. Int J Comput Assist Radiol Surg 19:241–251. 10.1007/s11548-023-03000-2 10.1007/s11548-023-03000-2 37540449

[18] Santoro-Fernandes V, Huff D, Scarpelli ML et al (2021) Development and validation of a longitudinal soft-tissue metastatic lesion matching algorithm. Phys Med Biol 66:155017. 10.1088/1361-6560/ac1457 10.1088/1361-6560/ac1457 PMC11329192 34261045

[19] Qahqaie M, Zimmer VA, Castaneda E et al (2025) Intelligent lesion selection: a novel method for longitudinal assessment of breast cancer lung metastases. In: Proceedings of medical imaging with deep learning (MIDL) 2025. Available via https://openreview.net/forum?id=Xk69ksan04 Accessed date: 11 Feb 2025

[20] Di Veroli B, Lederman R, Sosna J, Joskowicz L (2023) Graph-theoretic automatic lesion tracking and detection of patterns of lesion changes in longitudinal CT studies. In: Greenspan H, Madabhushi A, Mousavi P et al (eds) Proceedings of medical image computing and computer assisted intervention (MICCAI 2023). Springer Nature, Cham, pp 106–115

[21] Küstner T, Peisen F, Gatidis S et al (2025) Longitudinal-CT. University of Tübingen. Available via 10.57754/FDAT.qwsry-7t837 Accessed date: 05 May 2025

[22] Yang H, Schwartz LH, Zhao B (2016) A response assessment platform for development and validation of imaging biomarkers in oncology. Tomography 2:406–410. 10.18383/j.tom.2016.00223 10.18383/j.tom.2016.00223 PMC6037929 30042969

[23] Wasserthal J, Breit H-C, Meyer MT et al (2023) TotalSegmentator: robust segmentation of 104 anatomic structures in CT images. Radiol Artif Intell 5:e230024. 10.1148/ryai.230024 37795137 10.1148/ryai.230024 PMC10546353

[24] Czolbe S, Pegios P, Krause O, Feragen A (2023) Semantic similarity metrics for image registration. Med Image Anal 87:102830. 10.1016/j.media.2023.102830 37172390 10.1016/j.media.2023.102830

[25] Akiba T, Sano S, Yanase T et al (2019) Optuna: a next-generation hyperparameter optimization framework. In: Proceedings of the 25th ACM SIGKDD international conference on knowledge discovery & data mining. Association for Computing Machinery, New York, pp 2623–2631

[26] Bergstra J, Bardenet R, Bengio Y, Kégl B (2011) Algorithms for hyper-parameter optimization. In: Proceedings of the 25th international conference on neural information processing systems. Curran Associates Inc., Red Hook, pp 2546–2554

[27] Ma J, Yang H, Chou Y et al (2025) Generalizability of lesion detection and segmentation when ScaleNAS is trained on a large multi-organ dataset and validated in the liver. Med Phys 52:1005–1018. 10.1002/mp.17504 39576046 10.1002/mp.17504

[28] Karunanayake N, Lu L, Yang H et al (2025) Dual-stage AI model for enhanced CT imaging: precision segmentation of kidney and tumors. Tomography 11:3. 10.3390/tomography11010003 39852683 10.3390/tomography11010003 PMC11769543

