# Supplementary material for: Automated model-based lesion tracking in CT: colorectal liver metastases as a use case for development and performance analysis
Source: Eur Radiol Exp. 2026 Jul 14;10:107. doi: 10.1186/s41747-026-00750-x (PMC13369105; doi:10.1186/s41747-026-00750-x)
Supplement: Supplementary file 1 — Additional file 1: Table S1. CT acquisition and reconstruction characteristics for the internal MSKCC cohort and the external melanoma cohort. Table S2. Lesion annotation summary for the external validation dataset (melanoma patients, liver lesions). Table S3. Predictors for matched pair confidence. Table S4. Predictors for lonely lesion confidence. Table S5. Thresholds for Complex Event Identification. Table S6. Lesion-Level Precision, Recall, and F1-Score for the Proposed Affine + Auto MBT Versus Two Comparator Approaches, with Subgroup Analysis by Lesion Size and Total Intrahepatic Lesion Count (external melanoma dataset). Fig. S1. Auto-MBT model development pipeline. Fig. S2. Confusion matrices summarize lesion-level tracking performance across three algorithms (external melanoma dataset): (1) deformable + overlap, (2) deformable + Auto MBT, and (3) affine + Auto MBT (proposed). Rows denote ground truth (radiologist consensus); columns denote algorithm predictions. Performance is stratified across the full cohort, high tumor count cases (>10 lesions), and small lesion cases (<1 cm). Each cell reports absolute count and percentage of lesions in the corresponding category. The proposed method demonstrates substantially fewer misclassifications across all conditions. Fig. S3. (Top) Quantitative summaries plot lesion-level trajectories (longest diameter and volume) and compare baseline versus follow-up burden for target lesions and (bottom) for all lesions combined. Color categories are for visualization. Fig. S4. Complex lesion evolution—Merge illustrated in 3D and 2D. (Top) Two viewpoints show the baseline 3D map (multiple parent lesions BL10, BL19, BL20, BL22), the registered follow-up 3D map (matched lesion FU10), and the overlaid map demonstrating spatial continuity and preserved lesion neighborhood after affine liver-weighted registration. (Bottom) Registered axial CT slices (superior–inferior) depict the geometric correspondence between baseline and follow-up [file 41747_2026_750_MOESM1_ESM.pdf]

Automated model-based lesion tracking in CT: colorectal liver metastases as a use case for development and performance analysis

ELECTRONIC SUPPLEMENTARY MATERIAL

Table of contents

S1. Exclusion Criteria and Patient Selection for CRLM Cohort ………..…… Page 2

S1.1 CT acquisition and reconstruction characteristics ………………….….Page 2

S2. Consensus and Inter-Reader Agreement …………………………………..Page 2

S3. External Validation Melanoma Dataset Characteristics ………………….Page 3

S4. Liver Organ Segmentation and Affine Alignment …………………………Page 3

S5. Multi-cue Lesion Feature Extraction ……………………………………….Page 4

S6. Model Development Details ..……………………………………………….. Page 5

S7. Identification of Complex Lesion Events (Splits, Merges, and N↔M Complexes) ……………………………………………………………………………. Page 8

S8. Performance Metric Definitions and Statistical Testing ……………………Page 9

S9. Software and Libraries used to Develop the Tracking Pipeline …………Page 10

S10. Results ………………………………………………………………………Page 11

S11. Lesion Tracking Report……………………………………………………..Page 16

S12. Auto-MBT Faliure Case Analysis………………………………………..………………………Page 25

(S1) Exclusion Criteria and Patient Selection for CRLM Cohort

Exclusion criteria included incomplete liver coverage, non-contrast examinations, severe imaging artifacts, or substantial anatomical alterations (e.g., hepatic resections or advanced cirrhosis).

Consecutive adult patients diagnosed with unresectable CRLM who received first-line systemic chemotherapy between 2009 and 2019 at Memorial Sloan Kettering Cancer Center were retrospectively identified. Inclusion criteria were availability of two contrast-enhanced CT examinations: a baseline scan obtained immediately before treatment initiation and a follow-up scan performed approximately 8 weeks later, consistent with standard clinical evaluation intervals for systemic therapy response assessment. Exclusion criteria were incomplete liver coverage, non-contrast examinations, severe imaging artifacts, or substantial anatomical alterations (e.g., hepatic resections or advanced cirrhosis) that precluded reliable longitudinal lesion tracking. After applying these criteria, 87 patients were included, contributing 174 scans (87 baseline–follow-up pairs).

(S1.1) CT acquisition and reconstruction characteristics

Table S1. CT acquisition and reconstruction characteristics for the internal MSKCC cohort and the external melanoma cohort

Parameter	Internal MSKCC cohort	External melanoma cohort (public)
**Scanner manufacturer(s)**	GE MEDICAL SYSTEMS	Siemens
**Scanner model(s)**	LightSpeed VCT, LightSpeed1, Discovery CT750 HD	Sensation 64, SOMATOM Definition AS, SOMATOM Definition Flash, SOMATOM Force, Biograph128 PET/CT
**Acquisition protocol / patient position**	Whole body, supine, feet first	Whole-body staging protocol, supine, arms raised above head
**Contrast enhancement + phase**	contrast portal-venous phase	IV contrast, portal-venous phase
**Tube voltage (kVp)**	120	120
**Tube current**	range: 98-647 mA	Attenuation-based tube current modulation (CARE Dose, reference mA 240)
**Collimation (detector configuration)**	0.625 mm	64×0.6 mm or 128×0.6 mm
**Slice thickness (mm)**	2.5, 5.0	3.0
**Slice increment / spacing (mm)**	2.5, 5.0	3
**Reconstruction kernel**	STANDARD	Medium smooth kernel
**In-plane pixel spacing (mm)**	0.861 ± 0.092	0.754 ± 0.065

(S2) Consensus and Inter-Reader Agreement

Three radiologists independently (blinded) annotated lesion instance masks at baseline and follow-up CECT. Because instance IDs differed by reader, labels were harmonized per time point via overlap-based one-to-one assignment to form a common lesion index; unmatched components remained reader-unique. Consensus masks were generated per time point by voxel-wise majority voting (present if ≥2/3 readers).

Inter-reader agreement (n = 87). Agreement for lesion detection on the common index was summarized using percent three-reader concordance with 95% binomial CIs, Fleiss' κ (multi-rater), and pairwise Cohen's κ.

Baseline: 86.49% (95% CI, 85.44–87.53); Fleiss' κ = 0.7621 (substantial). Pairwise κ: R1–R2 0.6936 (substantial), R1–R3 0.8345 (almost perfect), R2–R3 0.7562 (substantial). Follow-up: 86.25% (95% CI, 85.15–87.35); Fleiss' κ = 0.7532 (substantial). Pairwise κ: R1–R2 0.7028 (substantial), R1–R3 0.8138 (almost perfect), R2–R3 0.7418 (substantial).

R1, R2, R3 denote Radiologists 1–3.

(S3) External Validation Melanoma Dataset Characteristics

Table S2. Lesion annotation summary for the external validation dataset (melanoma patients, liver lesions).

Characteristic	All patients (n = 23)
Lesion size distribution	
Baseline, n (%)	
< 1cm	101 (41.4%)
1-3 cm	117 (48.0%)
> 3 cm	26 (10.7%)
Lesion diameter (mean ± SD)	1.54 ± 1.34
Number of lesions per scan (mean ± SD)	10.61 ± 7.96
Follow-up, n (%)	
< 1cm	72 (44.4%)
1-3 cm	75 (46.3%)
> 3 cm	15 (9.3%)
Lesion diameter (mean ± SD)	1.53 ± 1.65
Number of lesions per scan (mean ± SD)	7.04 ± 3.91

Although whole-body CT scans were available[1], we analyzed only hepatic lesions (23 patients). The liver was segmented using TotalSegmentator[2], and analyses used liver-masked images.

In the CRLM test cohort, most lesions were ≥1 cm (Table 1), with only 12/276 (4.3%) baseline and 20/262 (7.6%) follow-up lesions <1 cm, while the majority measured 1–3 cm (69.6% baseline, 68.7% follow-up) or >3 cm (26.1% and 23.7%). Mean diameters were 2.51 ± 1.86 cm at baseline and 2.51 ± 1.74 cm at follow-up. In contrast, the melanoma validation cohort was dominated by smaller lesions (Table S2): 101/244 (41.4%) at baseline and 72/162 (44.4%) at follow-up were <1 cm, with few >3 cm (10.7% and 9.3%). Lesion diameters averaged ~1.5 cm at both timepoints. Thus, while CRLM lesions were measurable and spatially clustered (grouped), whereas melanoma lesions were often sub centimeter. Both datasets were challenging to track.

(S4) Liver Organ Segmentation and Affine Alignment

As a preprocessing step, we obtained liver masks using TotalSegmentator[2]. Masks were refined by hole filling and 10-mm dilation, then resampled to 1 × 1 × 1 mm (linear for images, nearest-neighbor for masks). Baseline and follow-up scans were cropped to the union liver bounding box with a ~20 mm margin.

Registration Framework: Follow-up images were registered to baseline using a 12-DOF affine transform (handles rotation, scaling, shearing, and translation).

$T(\mathrm{x}) = A\mathrm{x} + \mathrm{t}$,

Where $A \in \mathbb{R}^{3\times 3}$ and $\mathrm{t} \in \mathbb{R}^3$.

We optimized normalized cross-correlation on distance maps D derived from dilated liver masks.

Craniocaudal Weighting: The objective function incorporated spatial weighting along the superior-inferior axis to down-weight regions prone to diaphragmatic motion and segmentation uncertainties. The weight function $W(z)$ was defined as:

$$W(z) = \begin{cases} 0.3 + 0.7 \cdot 0.5\left(1 + \cos\left(\pi \cdot \left(1 - \frac{z}{0.2L}\right)\right)\right), & 0 \le z \le 0.2L \text{ (inferior 20\%)} \\ 1.0, & 0.2L \le z \le 0.8L \text{ (central 60\%)} \\ 0.3 + 0.7 \cdot 0.5\left(1 + \cos\left(\pi \cdot \left(1 - \frac{L - z}{0.2L}\right)\right)\right), & 0.8L \le z \le L \text{ (superior 20\%)} \end{cases}$$

Where L represents the total superior-inferior extent of the liver. This function assigns full weight (1.0) to the central 60% of the liver, with smooth cosine tapering to a minimum weight of 0.3 at the superior and inferior boundaries.

The weighted distance maps were computed as:

$$\widetilde{D}_{baseline} = D_{baseline} \cdot W, \widetilde{D}_{follow-up} = D_{follow-up} \cdot W$$

The optimal transformation was then obtained by maximizing:

$$T^* = \arg\max_T \mathrm{NCC}(\widetilde{D}_{baseline}, \widetilde{D}_{follow-up} \circ T)$$

Deformable Registration Baselines for Lesion Correspondence

For both deformable registration comparators, we used an intensity-based liver-masked affine with B-spline deformable registration. Briefly, an affine initialization is first estimated within the liver mask and then refined with a B-spline deformable transform optimized using Mattes mutual information, the resulting composite transform is applied to the follow-up scan and lesion masks before lesion correspondence is determined. In the deformable + overlap baseline, correspondences are defined using post-registration mask overlap, whereas in deformable + Auto-MBT the same registered masks are passed to Auto-MBT for multi-cue bipartite matching.

(S5) Multi-cue Lesion Feature Extraction

For each lesion pair (baseline lesion i, follow-up lesion j), we compute the following features:

1. Spatial Overlap (O) (Szymkiewicz-Simpson or Intersection-over-minimum)[3]

The overlap between corresponding lesion masks is measured using the intersection-over-minimum coefficient:

$$O_{ij} = \frac{\left|M_i^B \cap M_j^F\right|}{\min\left(\left|M_i^B\right|, \left|M_j^F\right|\right)},$$

where M_i^B and M_j^F are the binary masks of baseline lesion i and registered follow-up lesion j, respectively, and

|·| denotes the number of voxels. This measures containment and reaches 1 when one mask is fully contained in the other.

2. Centroid Proximity (D)

The Euclidean distance between lesion centroids in physical space:

$$d_{ij} = \left\|c_i^B - c_j^F\right\|_2, \; D_{ij} = \begin{cases} 0, d_{ij} > 80mm, \\ 1/(1 + \exp\left(\frac{(d_{ij} - 40)}{12}\right)), \text{otherwise.} \end{cases}$$

Where c_i^B and c_j^F are the 3D centroids of lesions i and j in physical coordinates. Distances beyond 80 mm are vetoed; closer lesions receive higher similarity.

3. Mean centered location (L)

Centroids were mean-centered within each scan to capture anatomical position relative to the liver:

$$r_i = c_i - \bar{c}, \quad \Delta r_{ij} = \left\|r_i^B - r_j^F\right\|_2,$$

Where r_i^B and r_j^F are the robust location vectors, comupted as, $r_i = c_i - \overline{c}$, with $\overline{c}$ being the mean centroid of all lesions in the respective scan. Differences were normalized to a [0,1] similarity using the 95th percentile of all pairwise Δr:

$$L_{ij} = 1 - \min\left(1, \frac{\Delta r_{ij}}{Q_{95}^{loc}}\right)$$

4. Nearest-Neighbor Shift (N)

For each lesion, the distance to its nearest neighbor within the same scan was computed (d_{nn}). Changes across timepoints were converted to similarity:

$$N_{ij} = \begin{cases} 1.0, & if\ d_{nn,i}^B = \infty \wedge d_{nn,j}^F = \infty, \\ 0.0, & if\ d_{nn,i}^B = \infty \oplus d_{nn,j}^F = \infty, \\ 1 - \min\left(1, \frac{\left|d_{nn,i}^B - d_{nn,j}^F\right|}{Q_{95}^{nn}}\right), & otherwise. \end{cases}$$

Both isolated lesions (no neighbors) are considered a perfect match $(N = 1)$; mismatch occurs if only one is isolated; otherwise, the absolute shift is scaled by the 95th percentile of observed differences.

(S6) Model Development Details

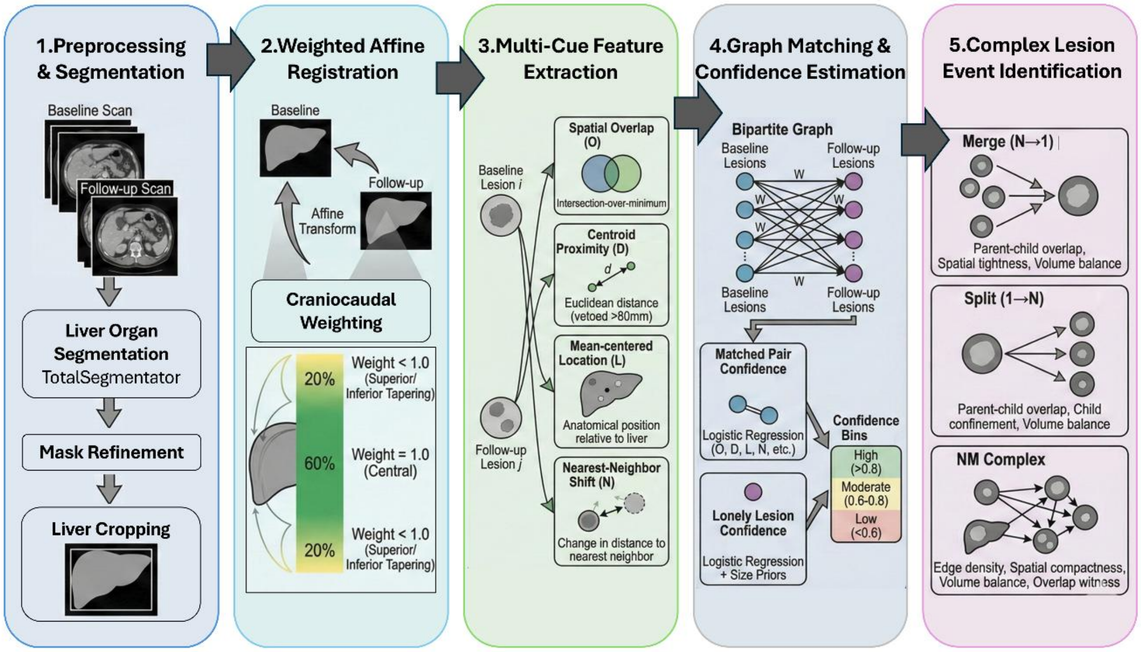

Figure S1. Auto-MBT model development pipeline.

(S6.1) Graph-Based Lesion Correspondence Framework

We formulate lesion tracking as a weighted bipartite graph $G = (\mathcal{B} \cup \mathrm{F}, E, W)$ where,

$\mathcal{B} = \{b_1, \ldots\ldots, b_n\}$ represents baseline lesions

$\mathrm{F} = \{f_{1,\ldots\ldots,f_n}\}$ represents follow-up lesions

$E \subseteq \mathcal{B} \times \mathrm{F}$ are edges between lesions

$W : E \rightarrow [0,1]$ assigns similarity weights

Comprehensive Similarity Function

Building on the extracted features from Feature Extraction, the similarity between baseline lesion i and follow-up lesion j is computed as:

$$S_{ij} = \widehat{\omega}_O . \tilde{O}_{ij} + \widehat{\omega}_D . \widetilde{D}_{ij} + \widehat{\omega}_L . \tilde{L}_{ij} + \widehat{\omega}_N . \widetilde{N}_{ij}$$

(S6.2) Cluster-Aware Matching

Cluster Detection: Build adjacency matrix A where, $A_{ij} = 1 \; if \; D_{ij} \leq 30 \text{ mm}$. Connected components with $|C| \geq 2$ from clusteres.

Linear arrangement: Via Principal Component Analysis (PCA) on cluster positions:

$$linearity = {}^{\lambda_1}/_{\lambda_2} > 2.5$$

(S6.3) Confidence Estimation

Matched Pair Confidence

For matched pairs (i, j), confidence is estimated using regularized logistic regression:

$$C_{match}(i,j) = \frac{1}{1 + \exp\left(-z_{match}\right)}$$

Where,

$$z_{match} = \beta_0 + \sum_{k=1}^{8} \beta_k \tilde{f}_k$$

With z-scored predictors $\tilde{f}_k$

Table S3. Predictors for matched pair confidence

Feature number	Predictor $\tilde{f}_k$	Definition
1	Combined multi-cue similarity	Weighted sum of O (overlap), D (centroid proximity), L (mean-centered location), N (nearest-neighbor shift)
2	Top-2 margin	Difference between best similarity and second-best candidate
3	Centroid distance	Euclidean centroid distance
4	Overlap coefficient	Intersection-over-minimum similarity
5	Location difference	Mean-centered location distance (95th percentile normalized)
6	NN shift	Nearest-neighbor distance shift
7	Neighborhood consistency	Coherence of local displacement field
8	Registration surrogate	Liver Dice similarity (global registration quality)

Lonely Lesion Confidence (Disappeared/New)

For unmatched lesions, confidence is estimated as:

$$C_{lonley}(i,j) = \frac{1}{1 + \exp\left(-z_{lonely}\right)} \, .P_{size}$$

Where,

$$z_{lonely} = \beta_0 + \sum_{k=1}^{6} \beta_k \tilde{f}_k$$

With z-scored predictors $\tilde{f}_k$

Size-based priors applied:

$$P_{disappear/new}(d) = \frac{1}{1 + \exp\left((d - 20)/5\right)}$$

where d is lesion diameter in mm.

Table S4. Predictors for lonely lesion confidence

Feature number	Predictor $\tilde{f}_k$	Definition
1	Max similarity	Highest similarity to any candidate (row or col)
2	Min centroid distance	Nearest centroid across time
3	Local density change	Ratio of lesions within 50 mm radius
4	Neighborhood consistency	Coherence of local displacement field
5	Top-K entropy	Entropy of normalized top-K candidate similarities
6	Registration surrogate	Liver Dice similarity (global registration quality)

All predictors were z-scored across the training set before model fitting. Logistic regression models were trained with L2 regularization; hyperparameters were chosen by cross-validation. Confidence values were interpreted as calibrated probabilities and used to assign lesions into high (≥0.8), moderate (0.6–0.8), or low (<0.6) confidence bins for radiologist triage.

(S7) Identification of Complex Lesion Events (Splits, Merges, and N↔M Complexes)

The multi-cue features defined above (overlap O_{ij}, centroid distance d_{ij}), and volumes V, are used to identify complex lesion behaviors beyind 1:1 matches. Three categories are modeled: merges (N→1), splits (1→N), and general N↔M complexes.
Since no manual ground truth exists for such events, thresholds for plausibility were tuned empirically on the development cohort (see Supplementary Table S5).

Merge Detection (N→1)

A follow-up lesion F_j may represent the merger of baseline lesions $\mathcal{P} = \{B_{p_1}, B_{p_2}, \ldots.\}$.

A merge is declared if:

1. Parent-child overlap
$$O_{Pj} \geq \theta_{\text{merge,ovlp}}, \;\; \forall p \in \mathcal{P}.$$
2. Spatial tightness of parents (parent centroids form a spatially compact cluster relative to local lesion spacing)
$$\max_{p,q \,\in \mathcal{P}} \left\| c_p^B - c_q^B \right\| \leq K_{\text{merge}} \cdot \text{median}\{d_{nn}(p)\}.$$
3. Volume balance (the combined baseline volume approximates the child volume within a tolerance band)
$$\delta_{\text{merge,low}} \leq \frac{\sum_{p \in \mathcal{P}} V_p^B - V_j^F}{\max\left(V_j^F, 10^{-6}\right)} \leq \delta_{\text{merge,high}}.$$

Split Detection (1→N)

A baseline lesion B_i may split into follow-up lesions $\mathcal{C} = \{F_{q_1}, F_{q_2}, \ldots.\}$.

A split is declared if:

1. Parent-child overlap
$$O_{iq} \geq \theta_{\text{split,ovlp}}, \quad \forall q \in \mathcal{C} .$$
2. Child confinement (child centroids lie within a confined region relative to the parent's physical extent)
$$\max_{p,r\,\in\,\mathcal{C}} \left\| c_q^F - c_r^F \right\| \leq K_{\text{split}} \cdot \text{eqDiam}(V_i^B), \qquad \text{eqDiam}(V) = \left(\frac{6V}{\pi}\right)^{1/3} .$$
3. Volume balance (the total child volume approximates the parent volume within tolerence)
$$\delta_{\text{split,low}} \leq \frac{\sum_{p\in\mathcal{C}} V_q^F - V_i^B}{\max(V_i^B, 10^{-6})} \leq \delta_{\text{split,high}}.$$

General N↔M Complexes

Remaining unmatched lesions are grouped into bipartite components $(\mathcal{B}, \mathcal{F})$ via similarity scores. A component is labeled a complex if:

1. Edge density (sufficient baseline–follow-up connections exist)
$$E_{\mathcal{G}} \geq \rho_{\min} \cdot \min\left(|\mathcal{B}|, |\mathcal{F}|\right) .$$
2. Spatial compactness (lesion centroids are spatially compact as a group)
$$\max_{m,n\,\in\,\mathcal{B}\,\cup\,\mathcal{F}} \|c_m - c_n\| \leq K_{\text{spatial}} \cdot \text{median}(d_{nn}).$$
3. Volume balance (total baseline and follow-up volumes are balanced within tolerance)
$$\frac{\left|\sum_{i\in\mathcal{B}} V_i^B - \sum_{j\in\mathcal{F}} V_j^F\right|}{\max\left\{\sum_{i\in\mathcal{B}} V_i^B, \sum_{j\in\mathcal{F}} V_j^F\right\}} \leq \delta_{\text{complex,vol}}.$$
4. Overlap witness (at least one baseline–follow-up pair demonstrates overlap)
$$\exists (i,j) \in \mathcal{B} \times \mathcal{F}: O_{ij} \geq \theta_{\text{complex,ovlp}}.$$

Table S5. Thresholds for Complex Event Identification

Symbol	Description	Value
$\theta_{\text{merge,ovlp}}$	Parent–child overlap (merge)	0.10
K_{merge}	Merge spatial tightness factor	1.2 × median NN
$\delta_{\text{merge,low}}, \delta_{\text{merge,high}}$	Merge volume balance band	[−0.30, +0.50]
$\theta_{\text{split,ovlp}}$	Parent–child overlap (split)	0.10
K_{split}	Child confinement factor	0.8 × eqDiam(parent)
$\delta_{\text{split,low}}, \delta_{\text{split,high}}$	Split volume balance band	[−0.40, +0.35]
$\rho_{\min}$	Minimum edge density (complex)	0.5
K_{spatial}	Spatial compactness factor (complex)	1.2 × median NN
$\delta_{\text{complex,vol}}$	Volume balance tolerance (complex)	≤ 0.30
$\theta_{\text{complex,ovlp}}$	Overlap witness (complex)	0.15

Note - Thresholds were tuned empirically on the development set, as no ground truth annotations for merges, splits, or N↔M complexes exist. Values were selected to balance sensitivity with plausibility when reviewed qualitatively by expert radiologists.

(S8) Performance Metric Definitions and Statistical Testing

Lesion tracking performance was evaluated at the lesion level, with consensus radiologist annotations serving as the reference standard. Metrics were calculated separately for matched, disappeared, and new lesions, as defined below.

Matched lesions were those present in both baseline and follow-up scans.

- True Positive (TP): Lesions correctly matched between scans.
- False Positive (FP): Lesions incorrectly identified as matched.
- False Negative (FN): True matches missed by the algorithm.

Disappeared lesions were those present in the baseline scan but absent in the follow-up scan.

- True Positive (TP): Correctly identified disappearances.
- False Positive (FP): Incorrectly classified disappearances.
- False Negative (FN): Missed disappearances.

New lesions were those absent in the baseline scan but present in the follow-up scan.

- True Positive (TP): Correctly identified new lesions.
- False Positive (FP): Incorrectly classified as new.
- False Negative (FN): Missed new lesions.

For each category, precision, recall, and F1-score were computed as:

$$Precision = \frac{TP}{TP+FP},$$

$$Recall = \frac{TP}{TP+FN},$$

$$F1 - Score = 2 \times \frac{Precision \times Recall}{Precision + Recall}$$

Matched-lesion F1 was the primary endpoint reported, for hypothesis testing, pairwise comparisons between algorithms were performed on recall within each lesion category. To account for multiple lesions per patient, inference was performed using a patient-cluster bootstrap: patients (scan pairs) were treated as the resampling unit, with 10,000 bootstrap replicates. In each replicate, lesion-level recall was recomputed for each algorithm, and the difference in recall between algorithms was calculated. The resulting bootstrap distribution was used to estimate 95% confidence intervals (2.5–97.5 percentiles) and two-sided *P*-values.

For subgroup analyses, stratification was performed by lesion size (<1 cm, ≤3 cm, all lesions) and intrahepatic tumor burden (≤5, 6–10, >10 lesions per scan). Three pairwise algorithm comparisons were conducted within each analysis family. *P*-values were adjusted for multiple comparisons using the Benjamini–Hochberg false discovery rate procedure, with adjusted $P<.05$ considered significant.

Importantly, all analyses were conducted at the lesion level; patients with more lesions therefore contributed proportionally more information to pooled estimates. This design reflects the increased clinical burden and tracking complexity associated with high lesion counts.

(S9) Software and Libraries used to Develop the Tracking Pipeline

All analyses were implemented in Python 3.9. The core libraries included:

1. Medical image I/O and processing: SimpleITK (2.x), NiBabel (3.x), scikit-image (0.18+), OpenCV (cv2)
2. Numerical and scientific computing: NumPy (1.21+), SciPy (1.7+), pandas (1.3+)
3. Machine learning / modeling: scikit-learn (0.24+) (for PCA and preprocessing)
4. Bayesian optimization: Optuna (2.x) with Tree-structured Parzen Estimator (TPE) sampling
5. Visualization and plotting: Matplotlib (3.4+), Seaborn (0.11+), Plotly (5.x)
6. Interactive demo: Streamlit (1.x), streamlit-drawable-canvas, Pillow (PIL)
7. Graph/diagram utilities: Graphviz

(S10) Results

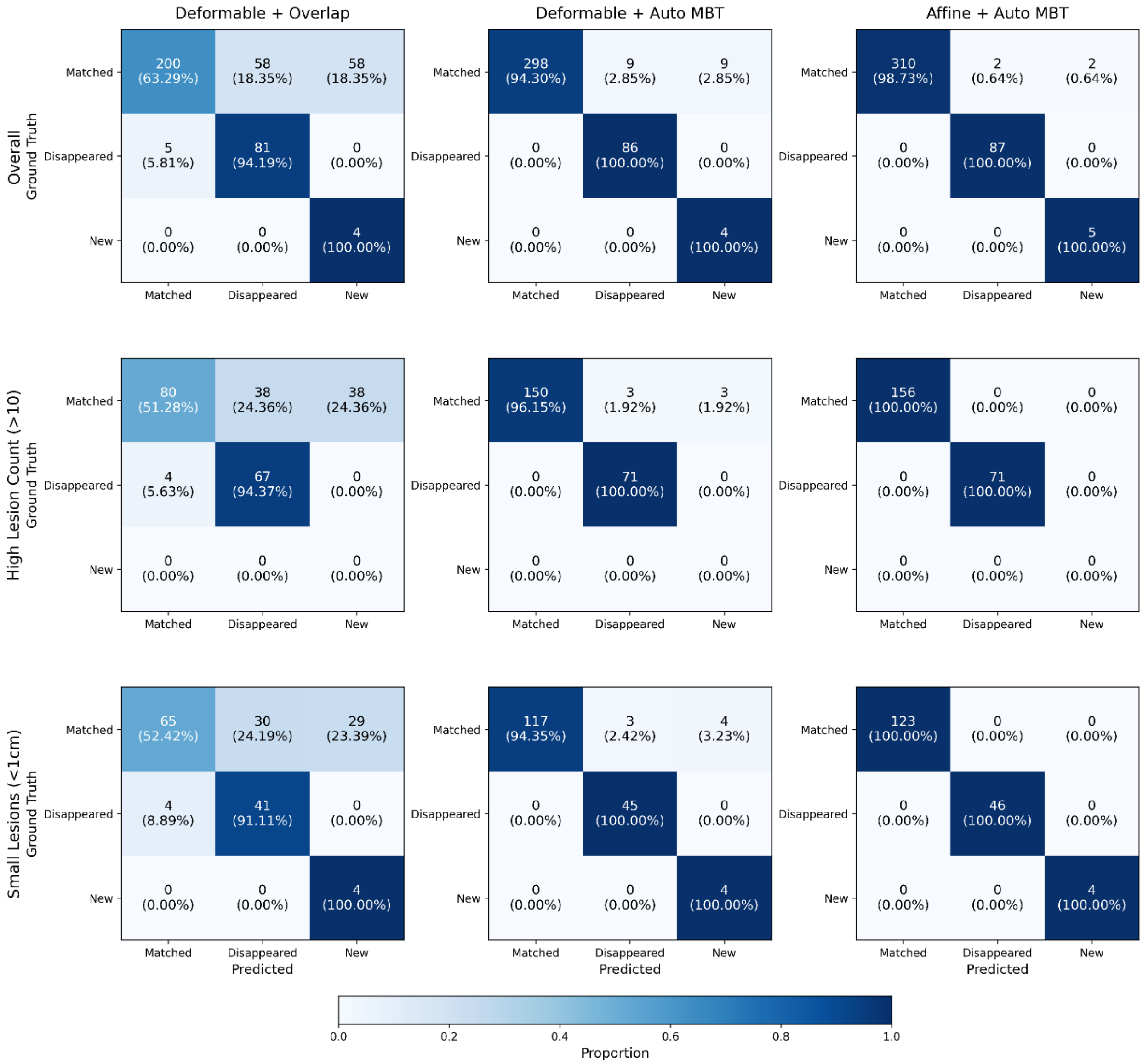

Figure S2. Confusion matrices summarize lesion-level tracking performance across three algorithms (external melanoma dataset): (1) deformable + overlap, (2) deformable + Auto MBT, and (3) affine + Auto MBT (proposed). Rows denote ground truth (radiologist consensus); columns denote algorithm predictions. Performance is stratified across the full cohort, high tumor count cases (>10 lesions), and small lesion cases (<1 cm). Each cell reports absolute count and percentage of lesions in the corresponding category. The proposed method demonstrates substantially fewer misclassifications across all conditions.

Table S6. Lesion-Level Precision, Recall, and F1-Score for the Proposed Affine + Auto MBT Versus Two Comparator Approaches, with Subgroup Analysis by Lesion Size and Total Intrahepatic Lesion Count (external melanoma dataset).

	**Metric**	Deformable + Overlap **Precision**	Deformable + Overlap **Recall**	Deformable + Overlap**F1**	Deformable + Auto MBT **Precision**	Deformable + Auto MBT **Recall**	Deformable + Auto MBT **F1**	Affine + Auto MBT **Precision**	Affine + Auto MBT **Recall**	Affine + Auto MBT **F1**	**P**(Deformable + Overlap vs Deformable + Auto MBT)	**P(**Deformable + Overlap vs Affine + Auto MBT)	**P(**Deformable + Auto MBT vs Affine + Auto MBT**)**
Overall Performance													
	Matched lesions	0.976 (0.944-0.990) [n=205]	0.633 (0.578-0.684) [n=316]	0.768 (0.705-0.820) [n=205]	1.000 (0.987-1.000) [n=298]	0.943 (0.912-0.964) [n=316]	0.971 (0.945-0.985) [n=298]	1.000 (0.988-1.000) [n=310]	0.987 (0.968-0.995) [n=314]	**0.994** (0.977-0.998) [n=310]	<0.001***	<0.001***	0.010**
	Disappeared lesions	0.583 (0.500-0.661) [n=139]	0.942 (0.871-0.975) [n=86]	0.720 (0.640-0.788) [n=139]	0.905 (0.830-0.949) [n=95]	1.000 (0.957-1.000) [n=86]	0.950 (0.886-0.979) [n=95]	0.978 (0.922-0.994) [n=89]	1.000 (0.958-1.000) [n=87]	**0.989** (0.939-0.998) [n=89]	0.007**	0.007**	1.000
	New lesions	0.065 (0.025-0.154) [n=62]	1.000 (n=4, CI unreliable)	0.121 (0.061-0.225) [n=62]	0.308 (0.127-0.576) [n=13]	1.000 (n=4, CI unreliable)	0.471 (0.239-0.716) [n=13]	0.714 (0.359-0.918) † [n=7]	1.000 (0.566-1.000) † [n=5]	**0.833** (0.464-0.967) † [n=7]	NA	NA	NA
By Lesion Size													
	Small lesions (<1cm)												
	Matched	0.942 (0.860-0.977) [n=69]	0.524 (0.437-0.610) [n=124]	0.674 (0.556-0.772) [n=69]	1.000 (0.968-1.000) [n=117]	0.944 (0.888-0.972) [n=124]	0.971 (0.923-0.989) [n=117]	1.000 (0.970-1.000) [n=123]	1.000 (0.970-1.000) [n=123]	1.000 (0.970-1.000) [n=123]	<0.001***	<0.001***	0.026*
	Disappeared	0.577 (0.462-0.685) [n=71]	0.911 (0.793-0.965) [n=45]	0.707 (0.593-0.800) [n=71]	0.938 (0.832-0.979) [n=48]	1.000 (0.921-1.000) [n=45]	0.968 (0.874-0.992) [n=48]	1.000 (0.923-1.000) [n=46]	1.000 (0.923-1.000) [n=46]	1.000 (0.923-1.000) [n=46]	0.023*	0.023*	1.000
	New	0.121 (0.048-0.273) [n=33]	1.000 (n=4, CI unreliable)	0.216 (0.110-0.382) [n=33]	0.500 (0.215-0.785)† [n=8]	1.000 (n=4, CI unreliable)	0.667 (0.339-0.886) † [n=8]	1.000 (n=4, CI unreliable)	1.000 (n=4, CI unreliable)	1.000 (n=4, CI unreliable)	NA	NA	NA
	Small-to-medium lesions (≤3cm)												
	Matched	0.971 (0.933-0.987) [n=170]	0.596 (0.537-0.652) [n=277]	0.738 (0.667-0.799) [n=170]	1.000 (0.985-1.000) [n=259]	0.935 (0.900-0.959) [n=277]	0.966 (0.937-0.982) [n=259]	1.000 (0.986-1.000) [n=271]	0.985 (0.963-0.994) [n=275]	0.993 (0.974-0.998) [n=271]	<0.001***	<0.001***	0.010**
	Disappeared	0.590 (0.505-0.669) [n=134]	0.940 (0.868-0.974) [n=84]	0.725 (0.644-0.793) [n=134]	0.903 (0.826-0.948) [n=93]	1.000 (0.956-1.000) [n=84]	0.949 (0.884-0.979) [n=93]	0.977 (0.920-0.994) [n=87]	1.000 (0.957-1.000) [n=85]	0.988 (0.938-0.998) [n=87]	0.007**	0.007**	1.000
	New	0.066 (0.026-0.157) [n=61]	1.000 (n=4, CI unreliable)	0.123 (0.062-0.228) [n=61]	0.308 (0.127-0.576) [n=13]	1.000 (n=4, CI unreliable)	0.471 (0.239-0.716) [n=13]	0.714 (0.359-0.918) † [n=7]	1.000 (0.566-1.000) † [n=5]	0.833 (0.464-0.967) † [n=7]	NA	NA	NA
	All lesions												
	Matched	0.976 (0.944-0.990) [n=205]	0.633 (0.578-0.684)	0.768 (0.705-0.820)	1.000 (0.987-1.000) [n=298]	0.943 (0.912-0.964) [n=316]	0.971 (0.945-0.985)	1.000 (0.988-1.000)	0.987 (0.968-0.995)	0.994 (0.977-0.998)	<0.001***	<0.001***	0.010**

			[n=316]	[n=205]			[n=298]	[n=310]	[n=314]	[n=310]			
	Disappeared	0.583 (0.500-0.661) [n=139]	0.942 (0.871-0.975) [n=86]	0.720 (0.640-0.788) [n=139]	0.905 (0.830-0.949) [n=95]	1.000 (0.957-1.000) [n=86]	0.950 (0.886-0.979) [n=95]	0.978 (0.922-0.994) [n=89]	1.000 (0.958-1.000) [n=87]	0.989 (0.939-0.998) [n=89]	0.007**	0.007**	1.000
	New	0.065 (0.025-0.154) [n=62]	1.000 (n=4, CI unreliable)	0.121 (0.061-0.225) [n=62]	0.308 (0.127-0.576) [n=13]	1.000 (n=4, CI unreliable)	0.471 (0.239-0.716) [n=13]	0.714 (0.359-0.918) † [n=7]	1.000 (0.566-1.000) † [n=5]	0.833 (0.464-0.967) † [n=7]	NA	NA	NA
By Tumor Burden													
	≤ 5 lesions												
	Matched	1.000 (0.898-1.000) [n=34]	0.810 (0.667-0.900) [n=42]	0.895 (0.749-0.960) [n=34]	1.000 (0.912-1.000) [n=40]	0.952 (0.842-0.987) [n=42]	0.976 (0.872-0.996) [n=40]	1.000 (0.912-1.000) [n=40]	1.000 (0.912-1.000) [n=40]	1.000 (0.912-1.000) [n=40]	0.683	0.683	0.683
	Disappeared	0.556 (0.267-0.811)† [n=9]	1.000 (0.566-1.000) † [n=5]	0.714 (0.395-0.905) † [n=9]	0.833 (0.436-0.970)† [n=6]	1.000 (0.566-1.000)† [n=5]	0.909 (0.509-0.990) † [n=6]	1.000 (0.610-1.000) † [n=6]	1.000 (0.610-1.000) † [n=6]	1.000 (0.610-1.000) † [n=6]	NA	NA	NA
	New	0.500 (0.215-0.785)† [n=8]	1.000 (n=4, CI unreliable)	0.667 (0.339-0.886) † [n=8]	0.800 (0.376-0.964)† [n=5]	1.000 (n=4, CI unreliable)	0.889 (0.453-0.987) † [n=5]	1.000 (0.566-1.000) † [n=5]	1.000 (0.566-1.000) † [n=5]	1.000 (0.566-1.000) † [n=5]	NA	NA	NA
	6-10 lesions												
	Matched	0.989 (0.938-0.998) [n=87]	0.729 (0.642-0.801) [n=118]	0.839 (0.748-0.902) [n=87]	1.000 (0.966-1.000) [n=108]	0.915 (0.851-0.953) [n=118]	0.956 (0.899-0.981) [n=108]	1.000 (0.967-1.000) [n=114]	0.966 (0.916-0.987) [n=118]	0.983 (0.939-0.995) [n=114]	0.007**	0.007**	0.828
	Disappeared	0.360 (0.202-0.555) [n=25]	0.900 (0.596-0.982) [n=10]	0.514 (0.330-0.695) [n=25]	0.667 (0.417-0.848) [n=15]	1.000 (0.722-1.000) [n=10]	0.800 (0.548-0.930) [n=15]	0.833 (0.552-0.953) [n=12]	1.000 (0.722-1.000) [n=10]	0.909 (0.637-0.983) [n=12]	0.828	0.828	1.000
	New	0.000 (0.000-0.194) [n=16]	0.000 (NA)	0.000 (0.000-0.194) [n=16]	0.000 (0.000-0.434)† [n=5]	0.000 (NA)	0.000 (0.000-0.434) † [n=5]	0.000 (n=2, CI unreliable)	0.000 (NA)	0.000 (n=2, CI unreliable)	NA	NA	NA
	> 10 lesions												
	Matched	0.952 (0.884-0.981) [n=84]	0.513 (0.435-0.590) [n=156]	0.667 (0.561-0.758) [n=84]	1.000 (0.975-1.000) [n=150]	0.962 (0.919-0.982) [n=156]	0.980 (0.943-0.993) [n=150]	1.000 (0.976-1.000) [n=156]	1.000 (0.976-1.000) [n=156]	1.000 (0.976-1.000) [n=156]	<0.001***	<0.001***	0.056
	Disappeared	0.638 (0.543-0.724) [n=105]	0.944 (0.864-0.978) [n=71]	0.761 (0.672-0.833) [n=105]	0.959 (0.887-0.986) [n=74]	1.000 (0.949-1.000) [n=71]	0.979 (0.916-0.995) [n=74]	1.000 (0.949-1.000) [n=71]	1.000 (0.949-1.000) [n=71]	1.000 (0.949-1.000) [n=71]	0.010**	0.010**	1.000
	New	0.000 (0.000-0.092) [n=38]	0.000 (NA)	0.000 (0.000-0.092) [n=38]	0.000 (n=3, CI unreliable)	0.000 (NA)	0.000 (n=3, CI unreliable)	0.000 (NA)	0.000 (NA)	0.000 (NA)	NA	NA	NA

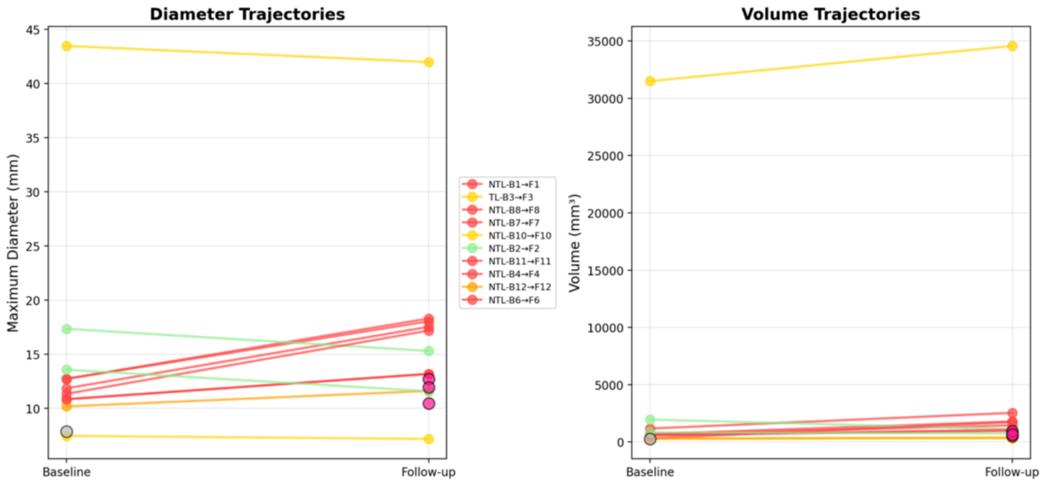

Longest diameter (mm): Baseline → Registered follow-up
and Volume (mm^3): Baseline → Registered follow-up.

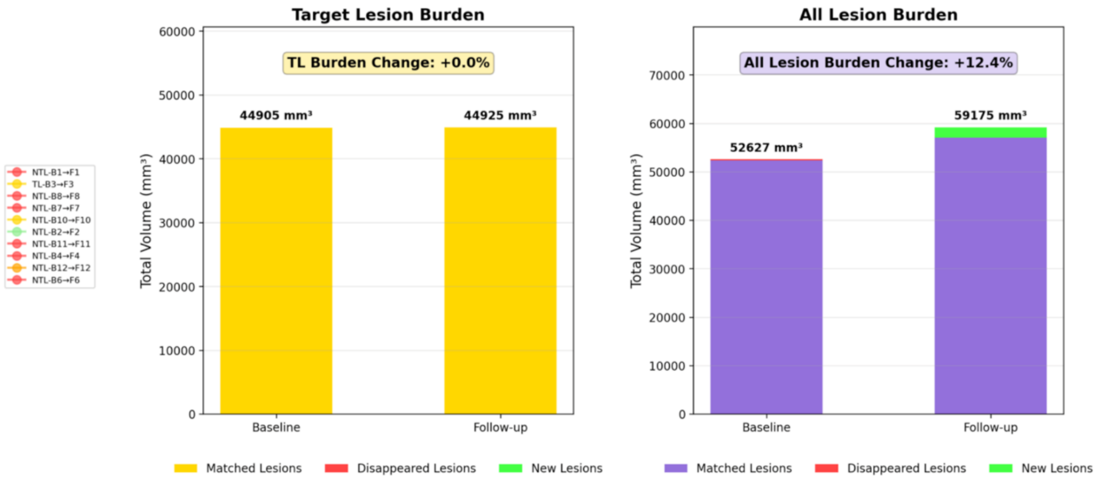

Target-lesion vs all-lesion burden (baseline– registered follow-up)

Figure S3. (Top) Quantitative summaries plot lesion-level trajectories (longest diameter and volume) and compare baseline versus follow-up burden for target lesions and (bottom) for all lesions combined. Color categories are for visualization.

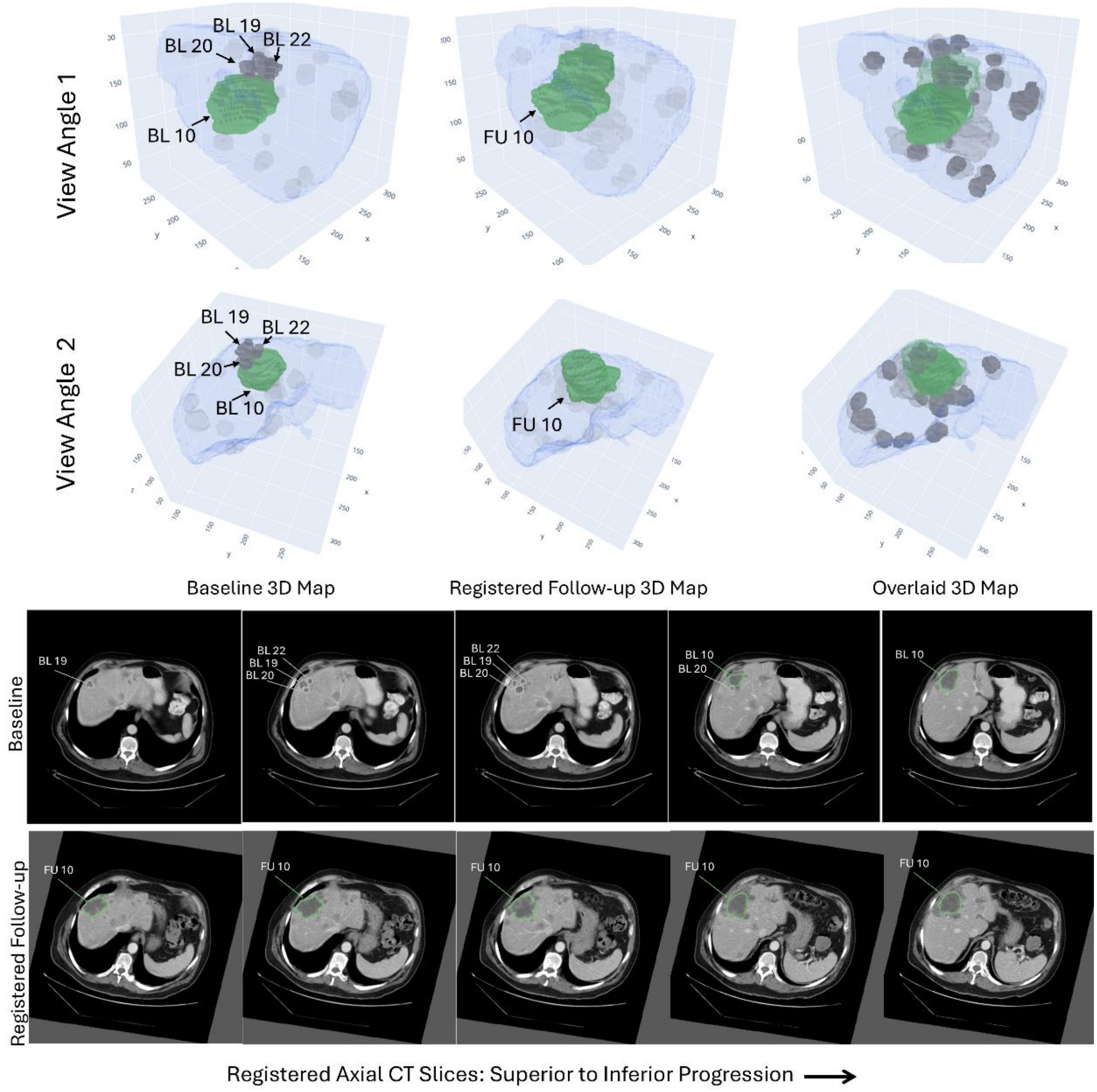

Figure S4. Complex lesion evolution—Merge illustrated in 3D and 2D.

(Top) Two viewpoints show the baseline 3D map (multiple parent lesions BL10, BL19, BL20, BL22), the registered follow-up 3D map (matched lesion FU10), and the overlaid map demonstrating spatial continuity and preserved lesion neighborhood after rigid liver-weighted registration. (Bottom) Registered axial CT slices (superior–inferior) depict the geometric correspondence between baseline and follow-up lesions, consistent with the 3D overlay; colors follow the response scheme in Fig 3.

(S11) Lesion Tracking Report

This is an example Lesion Tracking Report Generated by the interactive longitudinal lesion analysis tool (Affine + Auto MBT).

Longitudinal Lesion Tracking Report

**Patient ID:**	000175
**Data Path:**	Demo-01
**Report Date:**	2025-08-12 09:50
**Window Settings:**	C: 40, W: 400
**Total Lesions:**	16

Executive Summary

	Target Lesions	Non-Target Lesions	Total
**Baseline**	2	11	13
**Follow-up**	2	13	15
**Matched**	2	10	12
**Disappeared**	0	1	1
**New**	0	3	3

RECIST 1.1 Assessment

Stable Disease	2

Quantitative Analysis

Spider Plot - Diameter and Volume Trajectories

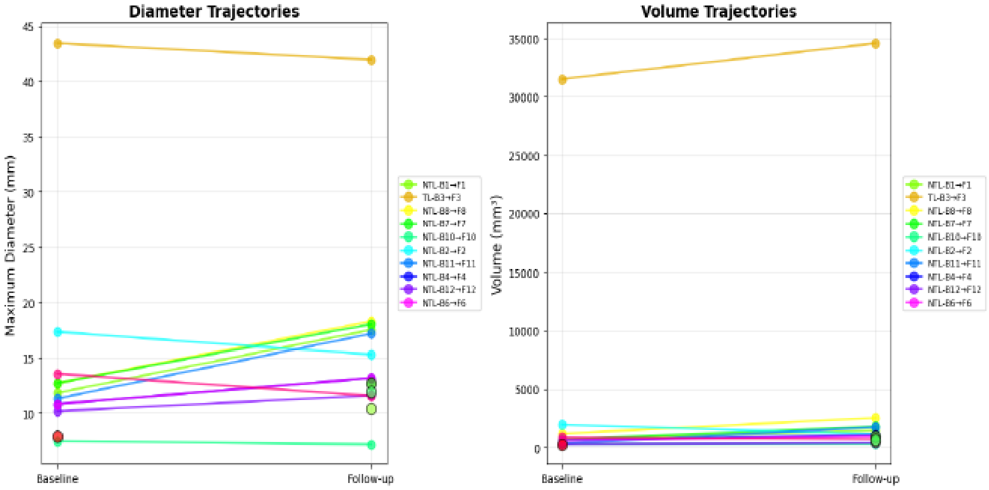

Tumor Burden Change Analysis

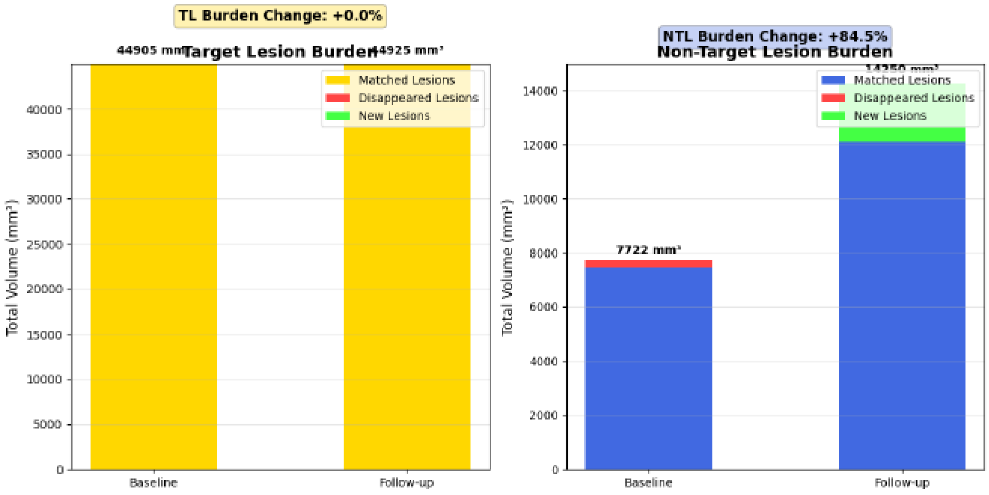

Detailed Lesion Information

ID	Category	Status	Confidence	Diameter Change	Volume Change
TL-B1	Target	Stable	97.4%	-20.9%	-22.7%
TL-B3	Target	Stable	95.3%	-3.4%	+9.7%
NTL-B1	Non-Target	Progressive	94.4%	+47.9%	+111.1%
NTL-B2	Non-Target	Regressive	91.3%	-11.8%	-42.4%
NTL-B4	Non-Target	Progressive	88.1%	+21.6%	+82.4%
NTL-B6	Non-Target	Progressive	82.8%	+21.6%	+59.4%
NTL-B7	Non-Target	Progressive	93.8%	+41.7%	+160.1%
NTL-B8	Non-Target	Progressive	95.1%	+43.8%	+116.9%
NTL-B9	Non-Target	Stable	71.5%	-14.7%	-13.9%
NTL-B10	Non-Target	Stable	92.3%	-3.8%	+4.6%
NTL-B11	Non-Target	Progressive	91.0%	+51.6%	+445.7%
NTL-B12	Non-Target	Progressive	83.2%	+14.1%	+35.4%
NTL-B13	Non-Target	Disappeared	65.3%	N/A	N/A
NTL-F14	Non-Target	New	90.4%	N/A	N/A
NTL-F15	Non-Target	New	92.0%	N/A	N/A
NTL-F16	Non-Target	New	87.9%	N/A	N/A

Individual Lesion Analysis

Each lesion shown at its maximum diameter slice

Target Lesions (2 total)

Lesion #1: TL-B1 - Status: Stable - Confidence: 97.4%

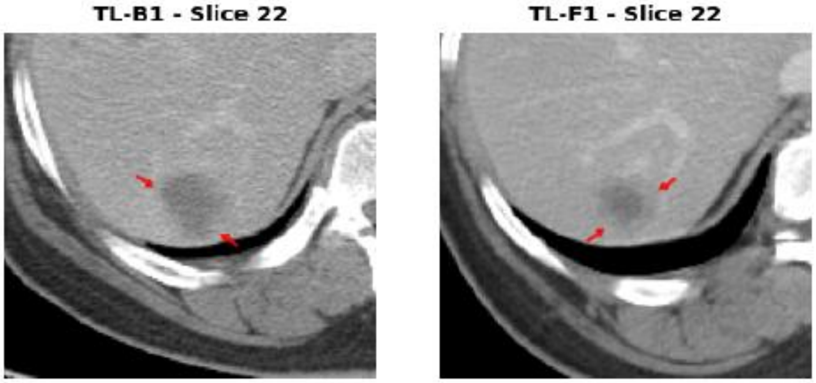

Baseline (Slice 22)
Diameter: 34.4
Volume: 13415.0

Follow-up (Slice 22)
Diameter: 27.2
Volume: 10365.0

Changes:
Diameter: -20.9%
Volume: -22.7%
RECIST: **SD**

Lesion #2: TL-B3 - Status: Stable - Confidence: 95.3%

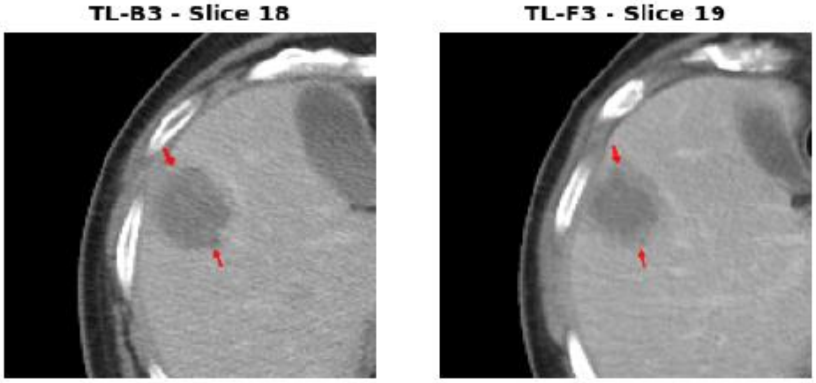

Baseline (Slice 18)
Diameter: 43.5
Volume: 31490.0

Follow-up (Slice 19)
Diameter: 42.0
Volume: 34560.0

Changes:
Diameter: -3.4%
Volume: +9.7%
RECIST: **SD**

Non-Target Lesions (14 total)

Lesion #3: NTL-B1 - Status: Progressive - Confidence: 94.4%

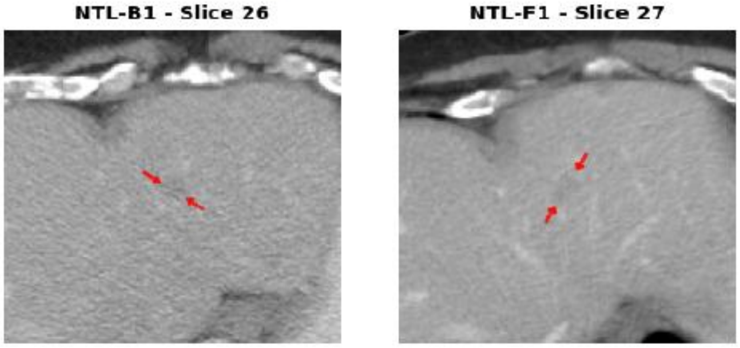

Baseline (Slice 26)	Follow-up (Slice 27)	Changes:
Diameter: 11.8	Diameter: 17.5	Diameter: +47.9%
Volume: 687.2	Volume: 1450.7	Volume: +111.1%

Lesion #4: NTL-B2 - Status: Regressive - Confidence: 91.3%

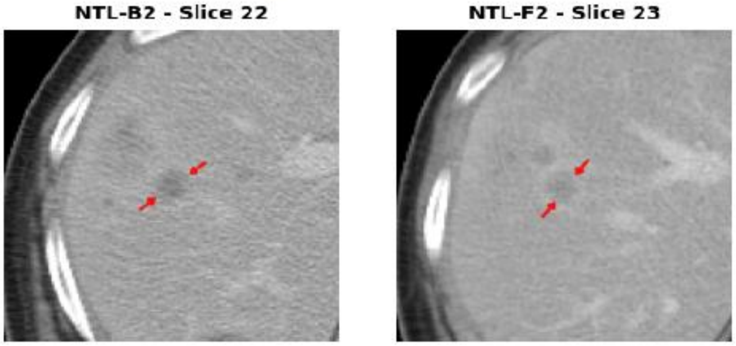

Baseline (Slice 22)	Follow-up (Slice 23)	Changes:
Diameter: 17.3	Diameter: 15.3	Diameter: -11.8%
Volume: 1957.4	Volume: 1127.9	Volume: -42.4%

Lesion #5: NTL-B4 - Status: Progressive - Confidence: 88.1%

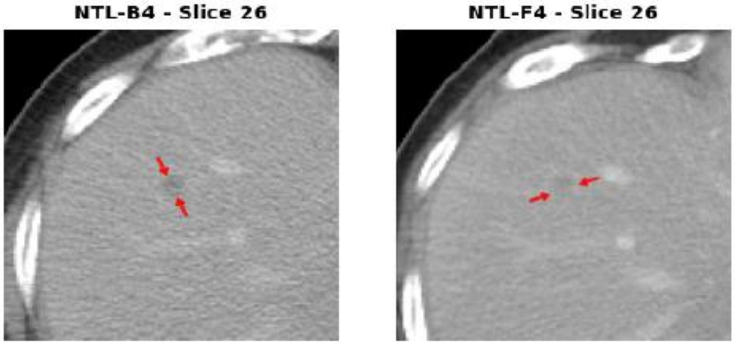

Baseline (Slice 26)	Follow-up (Slice 26)	Changes:
Diameter: 10.8	Diameter: 13.2	Diameter: +21.6%
Volume: 590.0	Volume: 1075.9	Volume: +82.4%

Lesion #6: NTL-B6 - Status: Progressive - Confidence: 82.8%

NTL-B6 - Slice 14 | **NTL-F6 - Slice 14**

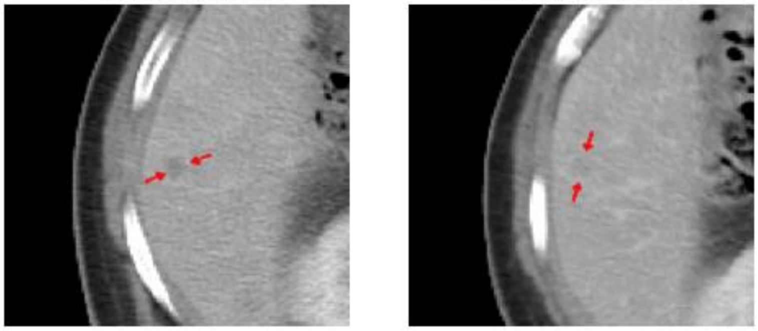

Baseline (Slice 14)
Diameter: 10.8
Volume: 607.3

Follow-up (Slice 14)
Diameter: 13.2
Volume: 968.3

Changes:
Diameter: +21.6%
Volume: +59.4%

Lesion #7: NTL-B7 - Status: Progressive - Confidence: 93.8%

NTL-B7 - Slice 13 | **NTL-F7 - Slice 13**

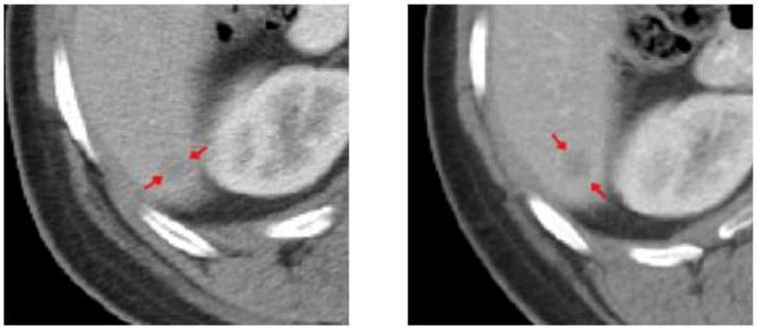

Baseline (Slice 13)
Diameter: 12.7
Volume: 687.2

Follow-up (Slice 13)
Diameter: 18.0
Volume: 1787.3

Changes:
Diameter: +41.7%
Volume: +160.1%

Lesion #8: NTL-B8 - Status: Progressive - Confidence: 95.1%

NTL-B8 - Slice 16 | **NTL-F8 - Slice 16**

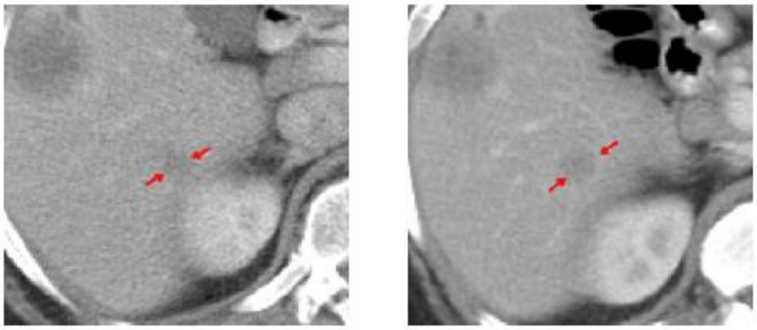

Baseline (Slice 16)
Diameter: 12.7
Volume: 1173.0

Follow-up (Slice 16)
Diameter: 18.3
Volume: 2543.9

Changes:
Diameter: +43.8%
Volume: +116.9%

Lesion #9: NTL-B9 - Status: Stable - Confidence: 71.5%

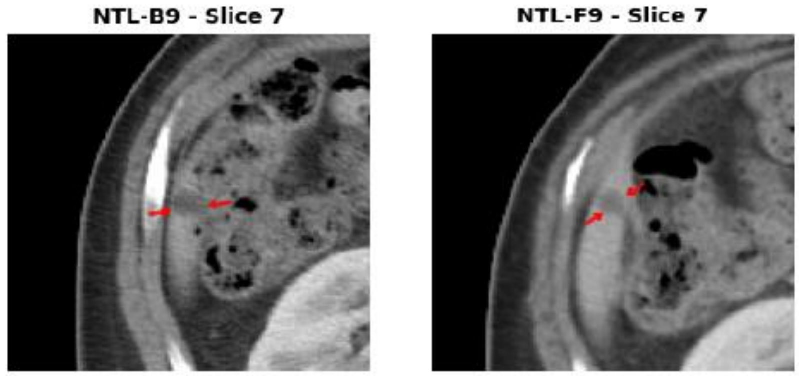

Baseline (Slice 7)
Diameter: 13.6
Volume: 846.8

Follow-up (Slice 7)
Diameter: 11.6
Volume: 728.8

Changes:
Diameter: -14.7%
Volume: -13.9%

Lesion #10: NTL-B10 - Status: Stable - Confidence: 92.3%

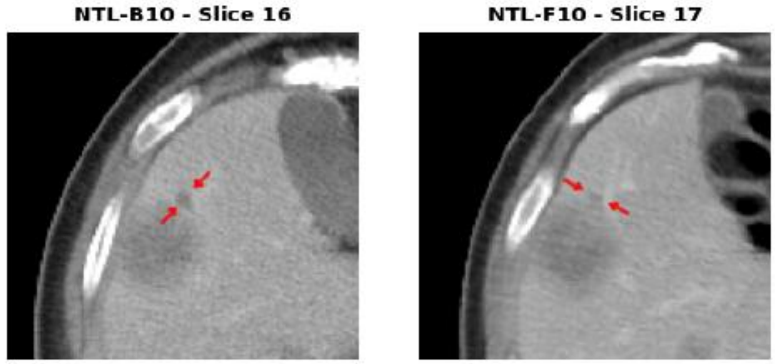

Baseline (Slice 16)
Diameter: 7.5
Volume: 301.9

Follow-up (Slice 17)
Diameter: 7.2
Volume: 315.8

Changes:
Diameter: -3.8%
Volume: +4.6%

Lesion #11: NTL-B11 - Status: Progressive - Confidence: 91.0%

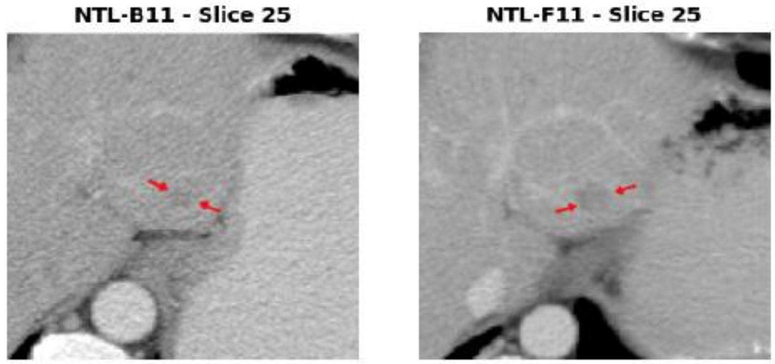

Baseline (Slice 25)
Diameter: 11.3
Volume: 319.3

Follow-up (Slice 25)
Diameter: 17.2
Volume: 1742.2

Changes:
Diameter: +51.6%
Volume: +445.7%

Lesion #12: NTL-B12 - Status: Progressive - Confidence: 83.2%

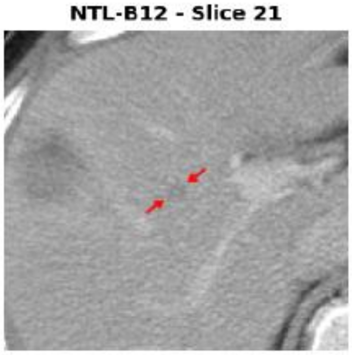

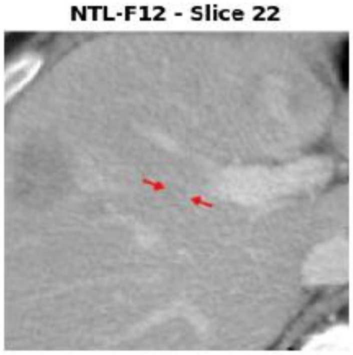

Baseline (Slice 21)
Diameter: 10.2
Volume: 284.6

Follow-up (Slice 22)
Diameter: 11.6
Volume: 385.2

Changes:
Diameter: +14.1%
Volume: +35.4%

Lesion #13: NTL-B13 - Status: Disappeared - Confidence: 65.3%

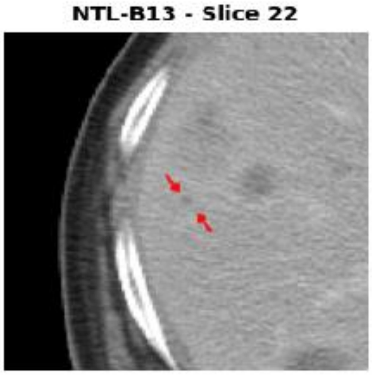

Baseline (Slice 22)
Diameter: 7.9
Volume: 267.2

Lesion #14: NTL-F14 - Status: New - Confidence: 90.4%

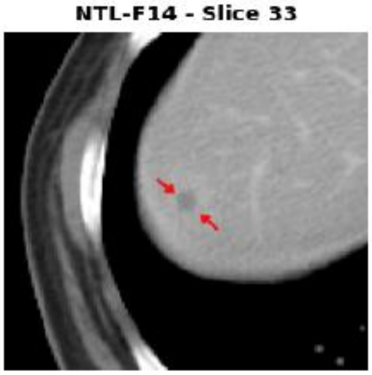

Follow-up (Slice 33)
Diameter: 12.7
Volume: 957.9

Lesion #15: NTL-F15 - Status: New - Confidence: 92.0%

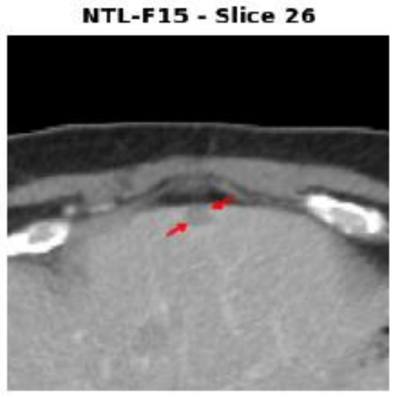

Follow-up (Slice 26)
Diameter: 10.4
Volume: 517.1

Lesion #16: NTL-F16 - Status: New - Confidence: 87.9%

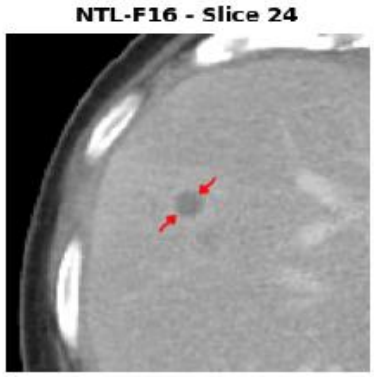

Follow-up (Slice 24)
Diameter: 11.9
Volume: 649.0

Report Summary

Total lesions analyzed: 16

Report generated on: 2025-08-12 09:50:13
Generated by: Longitudinal Lesion Tracking System

(S12) Auto-MBT Failure Case Analysis

Figures S5–S7 illustrate representative Auto-MBT correspondence failure modes observed in high lesion-burden examinations (≥10 lesions/scan). Errors occur most frequently in anatomically and technically challenging settings, including clusters of spatially proximate lesions, lesions abutting the liver capsule/boundary, and lesions with variable enhancement or interval morphologic change (e.g., hypervascular lesions). In some cases, residual misregistration after affine alignment under substantial inter-scan deformation further increases ambiguity for geometry-based matching.

For the 2D axial views, the liver contour is shown in yellow and lesion contours in red, annotated with their original lesion IDs. For clarity, we display only the lesions directly involved in the erroneous correspondence (other lesions are omitted in 2D). The accompanying 3D renderings include all lesions, with visual emphasis on the lesions involved in the error.

Failed case report #1

Figure S5 illustrates case 1, where ID switch in a clustered pericaval region

Reference standard (radiologist adjudication): Baseline lesion BL8 corresponds to follow-up lesion FU8.

Auto-MBT output: FU8 was incorrectly matched to BL17 (ID-switch).

Observation: Liver alignment was high (Dice = 0.93). The scan pair contained 18 baseline lesions and 11 follow-up lesions, with multiple lesions tightly clustered near the IVC, creating several plausible candidates with similar spatial/shape cues. Interval treatment-related change likely altered lesion morphology and local relative positions, increasing ambiguity for mask-based similarity metrics.

Confidence score: The (incorrect) association BL17->FU8 was assigned a low match confidence (0.521), indicating model uncertainty and supporting confidence-guided triage for radiologist review.

Interpretation: This error reflects a limitation of anatomy-agnostic, mask-driven correspondence in a crowded region where radiologists rely on stable local anatomic landmarks (e.g., relationship to the IVC and adjacent vascular structures) to preserve lesion identity.

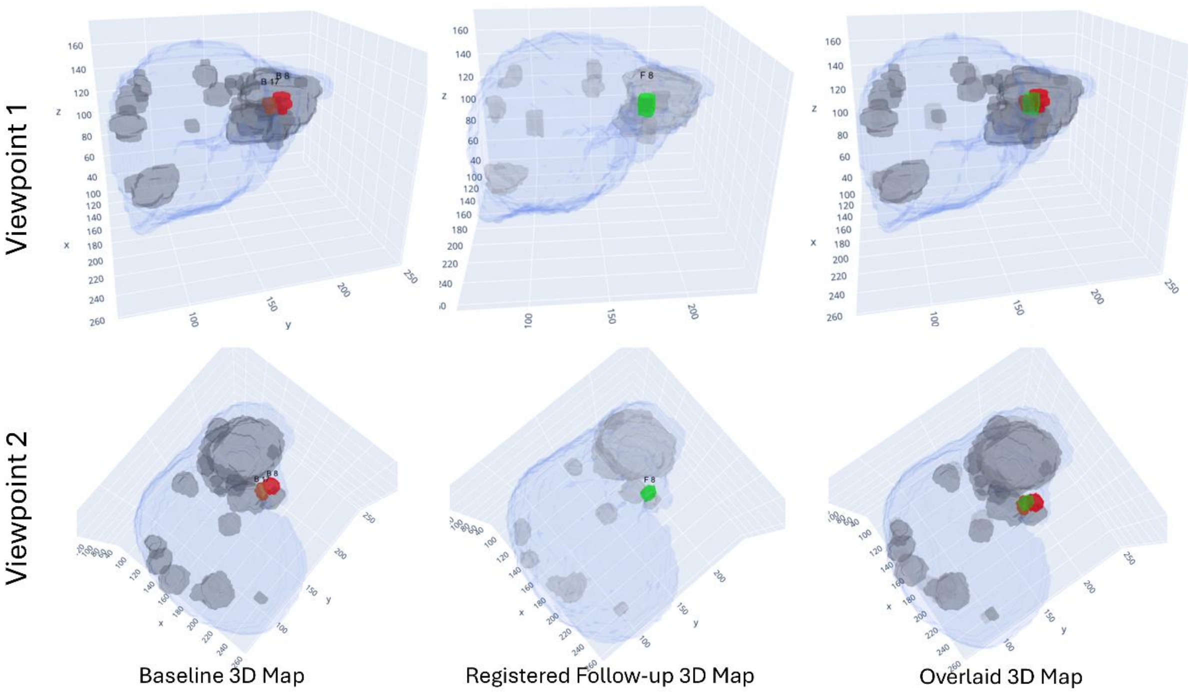

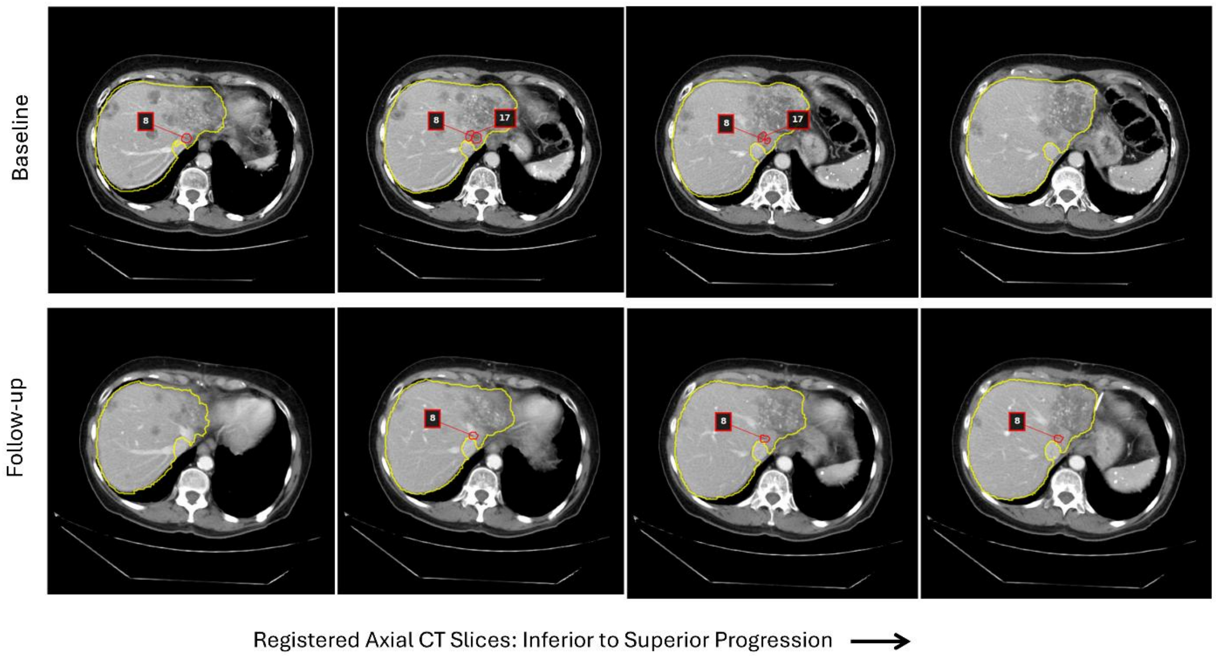

Figure S5. Auto-MBT failure case 1: ID-switch in clustered pericaval lesions

Failed case report #2

Figure S6 illustrates the ID-switch for boundary-adjacent lesions with residual cranio-caudal mismatch.

Reference standard (radiologist adjudication): Baseline lesion BL9 corresponds to follow-up lesion FU9.

Auto-MBT output: FU9 was incorrectly matched to BL19 (ID-switch).

Observation: Liver alignment was acceptable but imperfect (Dice = 0.855). The scan pair contained 24 baseline lesions and 13 follow-up lesions. The relevant lesions were near the liver boundary and partially clustered, where small residual alignment errors can produce disproportionate changes in centroid location and overlap. Inspection of the 3D rendering suggests that candidate lesions lie at slightly different cranio-caudal levels despite the overall liver Dice being >0.85, consistent with local residual misregistration and deformation plus interval changes.

Confidence score: The incorrect match BL19→FU9 carried low confidence (0.594), again indicating ambiguity and the need for radiologist verification.

Interpretation: Boundary-adjacent lesions are particularly sensitive to partial-volume effects, mask boundary jitter, and residual registration error, all of which can degrade the discriminative value of overlap/geometry cues and increase the risk of ID-switches.

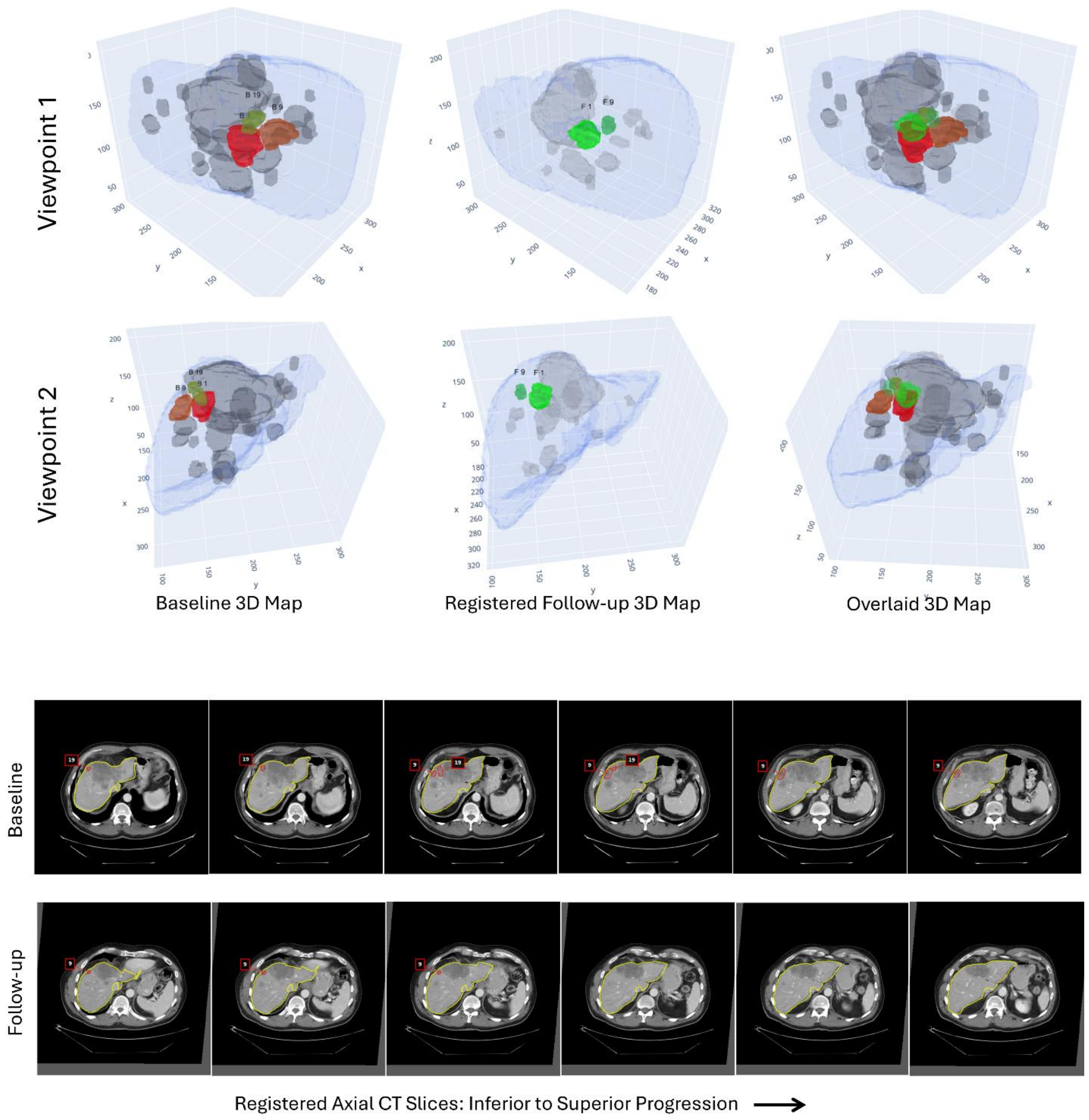

Figure S6. Auto-MBT failure case 2: ID-switch for boundary-adjacent lesions under residual misregistration

Failed case report #3

Figure S7 illustrates false disappeared/new assignment under marked interval deformation and morphologic change.

Reference standard (radiologist adjudication): Baseline lesion BL7 corresponds to follow-up lesion FU7.

Auto-MBT output: BL7 was labeled disappeared and FU7 was labeled new (missed correspondence).

Observation: Liver alignment was lower (Dice = 0.82) in this very high-burden case (31 baseline lesions, 22 follow-up lesions), with substantial interval change in liver shape (e.g., treatment effect and deformation). Under these conditions, affine alignment may leave clinically meaningful local residual misregistration, and lesion morphology/conspicuity may change enough that mask-based cues fail to link the correct pair.

Confidence score: Auto-MBT assigned low confidence to these status decisions (0.317 for BL7 and 0.273 for FU7), consistent with a highly ambiguous scenario that warrants clinician review.

Interpretation: Radiologists can often resolve such cases by anchoring lesion identity to local anatomic landmarks (e.g., fissures, vessel relationships, capsular proximity) across contiguous slices, contextual cues that are not explicitly available to a mask-only correspondence model.

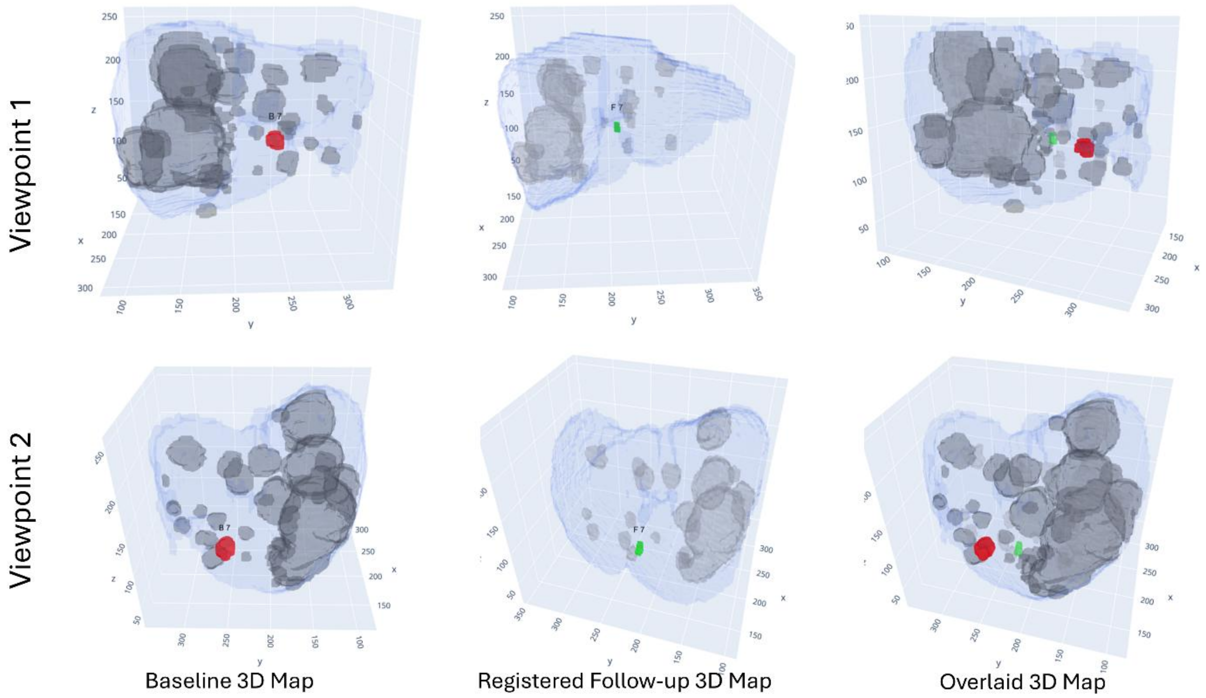

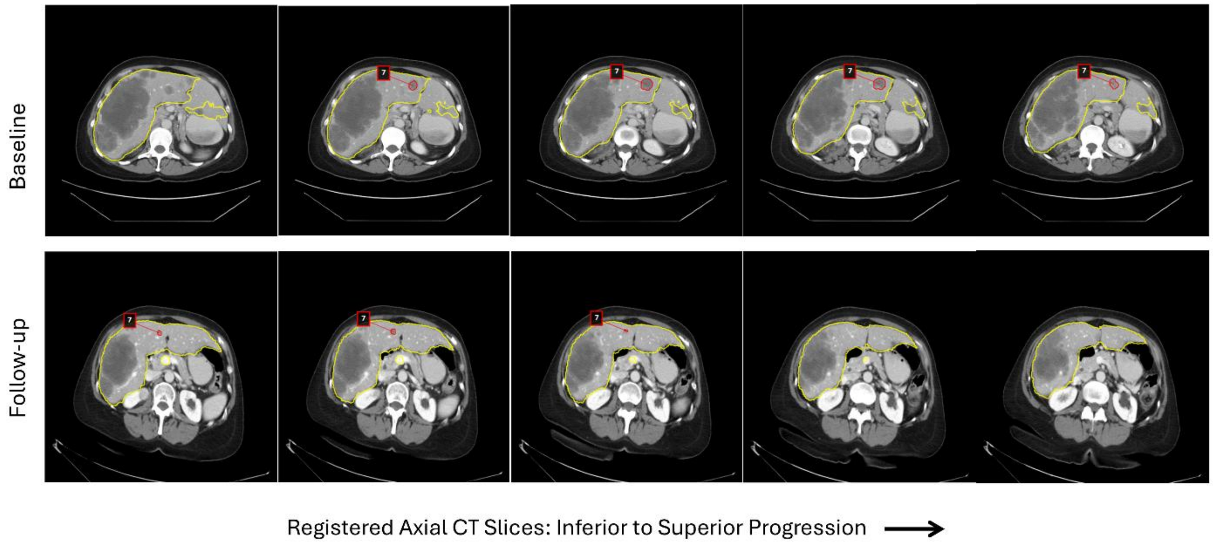

Figure S7. Auto-MBT failure case 3: Missed correspondence with false “disappeared/new” labels under marked interval deformation

Supplementary References

1. Küstner T, Peisen F, Gatidis S, et al. Longitudinal-CT. Published online March 16, 2025. doi:https://doi.org/10.57754/FDAT.qwsry-7t837

2. Wasserthal J, Breit HC, Meyer MT, et al. TotalSegmentator: Robust Segmentation of 104 Anatomic Structures in CT Images. *Radiol Artif Intell*. 2023;5(5):e230024. doi:10.1148/ryai.230024

3. Szymkiewicz D. Une conlribution statistique à la géographie floristique. *Acta Soc Bot Pol*. 2017;11(3):249-265. doi:10.5586/asbp.1934.012
